# Expert-guided approaches to complementary interventions for common side effects of cancer therapies: a practice-based perspective from integrative oncology centers in Baden-Württemberg, Germany

**DOI:** 10.3389/fonc.2025.1667298

**Published:** 2025-11-06

**Authors:** Marcela Winkler, Thomas Breitkreuz, Jürgen Brust, Stefanie Frenzel, Julia Gottfried, Wolfgang Heyl, Stefan Hiller, Ralf-Dieter Hofheinz, Meike Jocher, Elke Kaschdailewitsch, Hans Lampe, Maria Livas, Heike Mönnich, Claudia Raichle, Jane Reutter, Jens-Paul Seldte, Sigune Singer-Bayrle, Theresa Wagner, Anne-Kathrin Weise, Klaus Kramer

**Affiliations:** 1 Department of Integrative Medicin, Robert Bosch Hospital, Stuttgart, Germany; 2 Paracelsus-Krankenhaus Unterlengenhardt, Unterlengenhardt, Germany; 3 Diako Krankenhaus Mannheim, Mannheim, Germany; 4 Kreisklinikum Heidenheim, Heidenheim, Germany; 5 Klinik Öschelbronn, Niefern- Öschelbronn, Germany; 6 Department of Gynecologic Oncology, Regional Clinics Holding (RKH) Health Group Kliniken Ludwigsburg, Ludwigsburg, Germany; 7 Department of Integrative Oncology, Die Filderklinik, Filderstadt, Germany; 8 Department of Hematology and Oncology, University Medical Center Mannheim, Mannheim, Germany; 9 Oncology Center, Rems-Murr Klinikum Winnenden, Winnenden, Germany; 10 Departments of Gynecology and Internal Medicine III, Städtisches Krankenhaus Karlsruhe, Karlsruhe, Germany; 11 Department of Hematology, Oncology and Palliative Medicine, Klinikum Esslingen, Esslingen, Germany; 12 Tropenklinik Paul-Lechler-Krankenhaus Tübingen, Tübingen, Germany; 13 Department of Gynecologic, Regional Clinics Holding (RKH) Health Group Krankenhaus Bietigheim-Bissingen, Bietigheim-Bissingen, Germany; 14 Department of General and Visceral Surgery, Section Integrative Medicine, University Hospital Ulm, Ulm, Germany

**Keywords:** side effect, recommendations, expert consensus, integrative oncology, cancer therapy, mucositis, nausea, cancer-related fatigue

## Abstract

**Introduction:**

Cancer patients commonly suffer from substantial side effects of oncological therapies. Therefore, the Oncology Working Group of the Competence Network for Integrative Medicine in Baden-Württemberg, Germany (KIM-BW) developed practice-oriented recommendations for the integrative treatment of chemotherapy-induced mucositis (CIM), nausea and vomiting (CINV), and cancer-related fatigue (CRF).

**Methods:**

Two expert groups of physicians and nurses developed therapeutic recommendations using an interdisciplinary expert consensus process oriented on a Delphi-methodology with a standardized scoring matrix, considering training, feasibility, time intensity, clinical effectiveness, contraindications, and interactions. The consensus process was complemented by a targeted, non-systematic literature search conducted across the AWMF S3 Guideline on Complementary Medicine in Oncology, the KOKON knowledge database, the Working Group on Integrative Care in Oncology, and PubMed/Medline.

**Results:**

The expert panel consisted of 21 professionals (14 physicians, 7 nurses), all conventionally trained with additional qualifications in integrative disciplines. We evaluated 83 interventions. Top recommendations were identified for each symptom. For CIM: sage tea mouth rinses, ice cubes, sea buckthorn oil mouth rinses, frozen pineapple cubes, and herbal oral balm. For CRF: movement therapy, yarrow liver compresses, viscum album therapy, sleep hygiene with regular circadian rhythms, and hydrotherapy. For CINV: acupressure, ginger, aromatherapy, bitter botanicals such as gentian root, and homeopathic preparation *nux vomica*.

**Conclusions:**

Integrative treatment recommendations developed by the KIM Oncology Working Group provide pragmatic, clinically grounded guidance for integrative management of common treatment-related symptoms in oncology. Prospective evaluation of safety, effectiveness, and implementation across settings is warranted.

## 1 Introduction

At a global level, cancer remains the second leading cause of death after cardiovascular diseases. In 2023, there were an estimated 18.5 million new cancer cases worldwide and 10.4 million deaths, and 271 million disability-adjusted life years (DALYs) attributable to cancer. Projections suggest that by 2050, the global burden could reach 30.5 million new cases and 18.6 million deaths (1). The ‘Center for Cancer register data of the Robert Koch Institute’ reports that cancer causes the highest disease burden in Germany, with an estimated 500,000 new cancer cases annually and a five-year prevalence of approximately 1.64 million (2). Advances in cancer therapy have raised survival rates to nearly 80% in recent years; however, ensuring and enhancing quality of life (QoL) during and after treatment continues to be a critical challenge (3).

Patients undergoing cancer therapy often suffer from a wide range of side effects, necessitating effective symptom. Although advances in oncological therapies have improved survival rates, treatment-related side effects continue to represent a substantial burden for patients (4, 5). Some symptoms persist long-term, particularly CRF, sleep disturbances and anxiety, and may continue for up to ten years after curative therapy (4, 5). Oral CIM affects approximately 75% of patients receiving chemotherapy or radiotherapy (6), and CRF remains one of the most common and debilitating symptoms, reported in up to 70% of cancer patients (7). These symptoms are not only distressing on their own but also frequently co-occur, amplifying their negative impact on patients’ QoL (8). They remain among the most common and distressing side effects of cancer treatment, affecting up to 80% of patients receiving chemotherapy without adequate prophylaxis (9). Inadequate control of CINV can result in malnutrition, dehydration, electrolyte disturbances, treatment delays, and impaired QoL (9, 10). In the long term, unmanaged CINV may result in treatment delays, discontinuation of chemotherapy, and poor adherence to treatment plans—ultimately compromising therapeutic outcomes and reducing QoL (11).

To achieve adequate symptom relief, patients are increasingly turning to complementary approaches to manage side effects and enhance their overall well-being. The demand for evidence-based complementary therapies — particularly during and after cancer treatment — continues to grow. Worldwide, the use of integrative therapies has risen from an estimated 25% in the 1970s and 1980s to over 32% in the 1990s and 49% after 2000 (12). More recently, an international survey among oncology researchers and clinicians reported that more than half considered mind–body therapies the most promising category of integrative approaches in oncology (13). A multi-center cross-sectional survey conducted in 2021 in university hospitals in Baden-Württemberg, Germany, found that 48% of hospitalized patients were currently using complementary therapies. However, only 16% of patients had discussed this with their attending physician and over 80% wished for reliable information and for physicians to be better informed about complementary therapies (14). According to Jeitler et al. (2024), 70% of respondents in Germany indicated that they had used complementary methods during their lives, with 35% considering these as a supplement to conventional medicine, and 33% preferring an integrative combination of both approaches (15).

The increasing demand for integrative treatment approaches underscores the need for standardized therapeutic measures that are simple, cost-effective, and free of adverse effects. Integrative oncology, as defined by Witt et al. (2017), is a patient-centered, evidence-based area of cancer therapy that employs various methods such as mind-body techniques and natural products to optimize patients’ health and QoL (16). Moreover, according to the principles of evidence-based medicine defined by Sackett et al. (1996), clinical decisions should be made considering the best available scientific evidence, clinical expertise, and patient needs (17). This framework guides our group’s efforts to generate evidence-based recommendations for clinical practice.

Given the high utilization of integrative therapies among cancer patients and the growing body of supporting evidence, guidelines have been developed by organizations such as the Society for Integrative Oncology (SIO), the American Society of Clinical Oncology (ASCO), and other groups like the Gynecological Oncology Working Group (AGO) and the National Comprehensive Cancer Network (NCCN). Additionally, the S3 guidelines for complementary medicine in the treatment of cancer patients published by the German Cancer Society provide a comprehensive set of evidence-based recommendations for addressing side effects of cancer therapy (18). Recent trials, meta-analyses, and guideline updates support a broad range of integrative approaches in oncology. Representative examples include exercise, yoga and Qi Gong for CRF and for improvements in QoL (19–21), acupressure and acupuncture for CINV and CRF (22, 23), aromatherapy for CINV (24) and anxiety (25), mistletoe (viscum album) for improvements in QoL (18) and CRF (26), and Mind Body Medicine (MBM) Interventions, particularly for the management of CRF, anxiety, sleep disturbances, and emotional well-being (27). Furthermore, nursing-based integrative interventions play an important role in oncology care (28, 29).

However, practical challenges arise in everyday patient care regarding the implementation of these recommendations, mainly due to a lack of funding and structural support. Patients also face significant difficulties due to the burden of symptoms and the lifestyle changes imposed by cancer treatment. Supportive services, especially those that empower and motivate patients to take an active role in their health, are essential complements to oncology center interventions.

In summary, integrating complementary therapies into cancer treatment not only improves QoL but also optimizes treatment outcomes and can potentially reduce patient care costs (30). Promoting self-help strategies is important as patients actively seek ways to improve their health and alleviate their symptoms. The primary aim of this study was to develop integrative treatment recommendations for managing the five best interventions for three important symptoms associated with cancer therapies: CIM, CRF, and CINV.

## 2 Materials and methods

### 2.1 Design

The presented statement is based on an interdisciplinary consensus process followed by a complementary, targeted non-systematic literature search. This study was conducted within the Competence Network for Integrative Medicine in Baden-Württemberg (KIM–BW). Two professional expert groups – one composed of physicians and one of nursing professionals – were built from various clinical institutions across Baden-Württemberg (see. **Supplementary Table 1**) to develop evidence-informed treatment recommendations for three common symptoms related to cancer therapy: CIM, CRF, and CINV.

**Figure 1:**
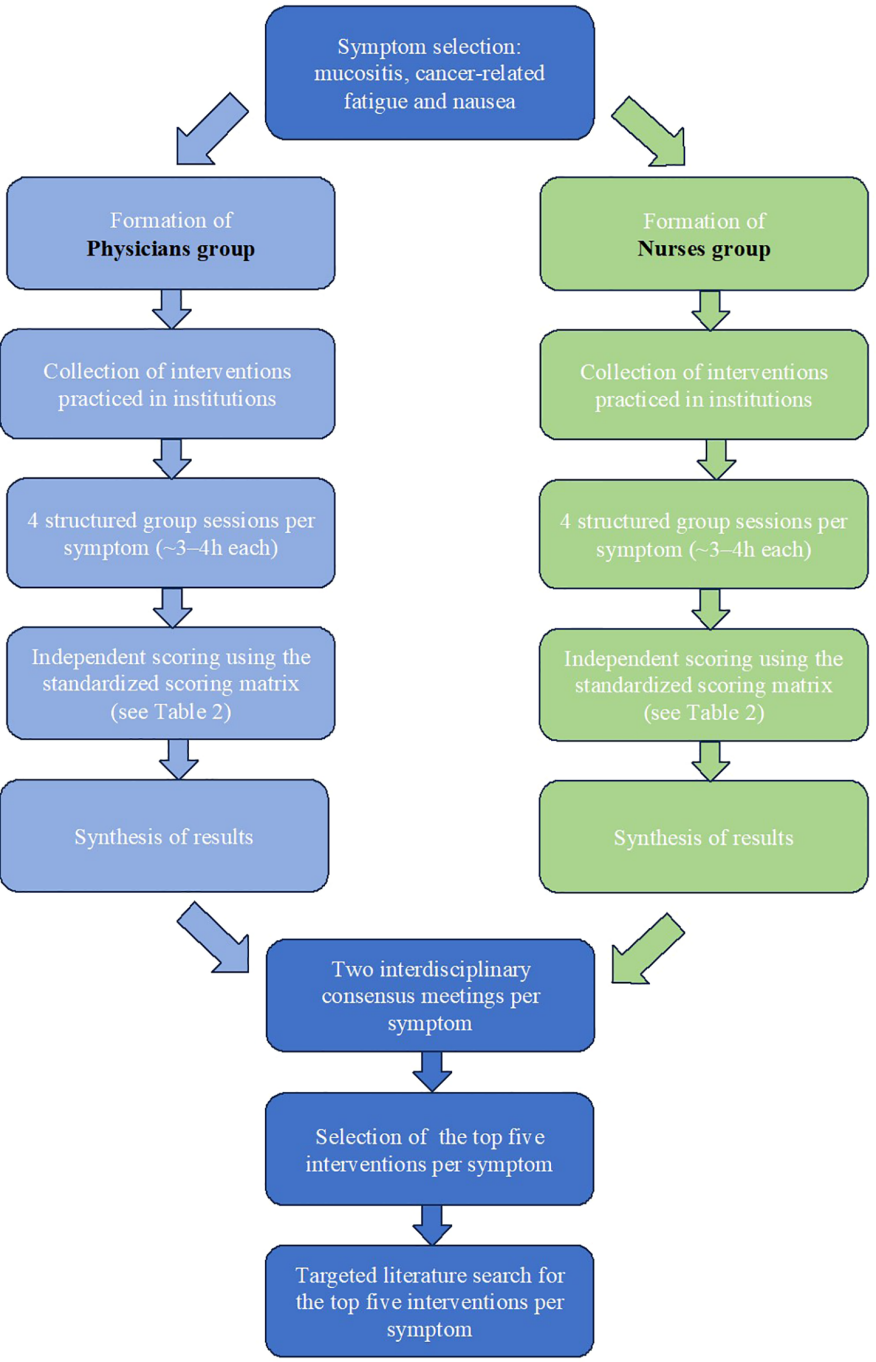
Flowchart consensus process.

The Delphi method is widely recognized as a structured, iterative process for achieving expert consensus through independent rating and controlled feedback (31). It is particularly useful for developing practice recommendations in areas with limited or heterogeneous evidence (31). This project applied a structured expert consensus process oriented on a Delphi-methodology, tailored to the practical context of integrative oncology (see **Figure 1**). Open, non-anonymous discussions were intentionally included to ensure clarity regarding the specific variations of non-standardized interventions. The process included multiple phases: identification of commonly used interventions, structured evaluation using predefined criteria as well as joint interdisciplinary meetings to consolidate findings and define final best-practice recommendations.

Detailed descriptions of the evaluation criteria, the scoring system, and consensus-building approach are provided in Sections 2.3, with full scoring definitions available in **Table 1**. Finally, a targeted non-systematic literature search was conducted in specialized databases (PubMed, KOKON, AWMF-S3 guidelines, among others) to support the consensus-based decisions. Although not systematic, this search identified relevant studies and clinical guidelines that partially support some of the selected interventions.

**Table 1:** Scoring matrix for evaluation of interventions (physician and nursing groups).

Category	Description	Score
Required Training (1)	Implementation possible with written instruction (e.g., information leaflet)	1
	Implementation after verbal instruction or demonstration is possible	2
	In-house training or continuing education required	3
	Specialized certification or restricted scope of practice required	4
	Advanced training with institutional cost or certification	5
Practical Feasibility (2)	Feasible through verbal recommendation, also by phone	1
	Requires prescription/order via visit or physician coordination	2
	Needs direct implementation by trained personnel (e.g., acupuncture)	3
	Requires specific materials or settings (e.g., treatment room)	4
Time Effort (3)	< 15 minutes	1
	< 30 minutes	2
	30–45 minutes	3
	45–60 minutes	4
	> 60 minutes	5
Effectiveness (4)	No symptom relief	1
	Slight symptom relief	2
	Clear symptom relief	3
	Reliable, marked symptom relief	4
	Sustained, reliable, significant symptom remission	5
Institutional Use(n/total)	Indicates the number of institutions currently applying the intervention out of the total number of institutions participating in the consensus process.	
Contraindications	Relevant risks and contraindications	
Interactions	Relevant interactions	
Special Notes (N, Pr, T)	Any important comments from experience or implementation practice Indicate if the intervention is used preventively (Pr), therapeutically (T).	

Both the physician and nursing expert groups used this matrix during the evaluation phase. Parameter (1), (2), (3), (4) were scored on a 5-point scale unless otherwise noted.

The scope of this consensus was limited to interventions routinely applied in European integrative oncology. As the group did not include specialists in Ayurveda or traditional Chinese herbal medicine, these approaches were not assessed. Acupuncture and acupressure were included as they are well established in clinical practice in Europe.

### 2.2 Participating institutions and experts

The expert groups included professionals from multiple clinical institutions across Baden-Württemberg, Germany with extensive experience in both conventional and integrative oncology. Participating institutions comprised hospitals and departments with long-standing practice in complementary therapies, such as the Robert Bosch Hospital, Stuttgart, Germany; Paracelsus-Krankenhaus Unterlengenhardt, Germany; Diako Krankenhaus Mannheim, Germany; Kreisklinikum Heidenheim, Germany; Klinik Öschelbronn, Germany; RKH Kliniken Ludwigsburg, Germany; Die Filderklinik, Filderstadt, Germany; University Medical Center Mannheim, Germany; Rems-Murr Klinikum Winnenden, Germany; Städtisches Krankenhaus Karlsruhe, Germany; Klinikum Esslingen, Esslingen, Germany; Paul-Lechler Krankenhaus Tübingen, Germany; RKH Krankenhaus Bietigheim-Bissingen, Germany, and the Department of General and Visceral Surgery, Section Integrative Medicine, University Hospital Ulm, Germany. Additionally, the Institute for General Practice and Interprofessional Care at the University Hospital Tübingen, Germany contributed to the process through literature research.

In total, 21 professionals (14 physicians and 7 nurses) from 14 clinical institutions participated in the consensus processes. Each institution delegated a team of professionals trained in conventional medicine or nursing with additional qualifications in various fields of complementary and integrative medicine (CIM). These included classical Traditional European Medicine (TEM) such as hydrotherapy, exercise therapy, nutrition therapy, phytotherapy, and lifestyle regulation, as well as aromatherapy, mind-body medicine such as yoga, mindfulness-based stress reduction (MBSR), mindful self-compassion (MSC), Qi Gong, traditional Chinese medicine including acupuncture and acupressure, homeopathy, and anthroposophic medicine. Nursing-based therapies such as compresses, effusions, and wraps were also represented. Ayurveda was not represented within the group, as no panelist had formal qualifications in this field. **Supplementary Table 1** summarizes details regarding the academic background, medical specialization, complementary medicine qualifications, and years of experience of each participant.

In addition to the listed participants, further members of the KIM-BW network contributed indirectly to the development of these recommendations. The appointed representatives from each participating institution consulted with their respective clinical teams to collect input and practical insights. This internal exchange ensured that the final recommendations incorporated the collective experience and routine practices of each institution, even beyond the listed participating experts.

### 2.3 Consensus process

The development of treatment recommendations resulted in a structured consensus process oriented on a Delphi-methodology for each selected symptom: CIM, CRF, and CINV. This process involved both expert groups, one of physicians and one of nursing professionals, who worked independently and collaboratively under the coordination of the KIM-BW team. **Supplementary Table 2** shows the participation by each institution and individual in the consensus process for each symptom. In the first phase, each professional group compiled a list of interventions currently practiced at their institutions. Each group held multiple structured sessions per symptom (typically four, 4 hours each), depending on the number of therapeutic options and the complexity of the procedures. The goal of these meetings was to establish a shared understanding of the interventions, including their specific procedures, which was essential due to non-standardized variations.

In the second phase, the groups rated each intervention independently using a standardized scoring matrix (see **Table 1**) developed and piloted by the coordination team based on a methodology previously applied by Stolz et al. (2021) (32), Steinmann et al. (2021) (6) in a similar consensus process oriented on a Delphi-methodology for nursing interventions in oncology. The matrix included four core parameters: 1) required training, 2) feasibility in routine clinical practice, 3) time requirements, and 4) estimated clinical effectiveness based on practical experience. Each parameter was scored on a 5-point scale with clearly defined anchors. Additionally, contraindications, interactions, the number of institutions applying the intervention, the classification as either preventive (Pr) or therapeutic (T) and special notes were documented separately by symptom.

The coordination team synthesized the results from both groups. Interventions that were represented in both groups (nurses and physicians) showed a low risk, a high perceived likelihood of symptom improvement, and were easy to implement were selected for evaluation, following a procedure oriented on a Delphi-methodology, but not adhering to a formal Delphi process. Unlike the classical Delphi design, we did not use anonymous rating rounds with controlled statistical feedback. Instead, consensus was reached in structured open discussions and by majority agreement after at least two rounds.

Exceptions were made for interventions that did not fully meet these criteria —such as those with a high number of interactions —but were included due to other factors, particularly their clinical relevance and frequent use in practice. All participants approved the summary list. The interventions for CIM listed by physicians can be found in **Supplementary Table 3**, those by nurses in **Supplementary Table 4**, physicians’ interventions for CRF in **Supplementary Table 5**, nurses’ interventions for CRF in **Supplementary Table 6**, physicians’ interventions for CINV in **Supplementary Table 7**, and nurses’ interventions for CINV in **Supplementary Table 8**. In the next phase both groups were represented by experts from institutions actively applying the respective interventions. Consensus meetings—two per symptom, each lasting two hours—were held to clarify open questions, align perspectives, and finalize the selection of the five best-practice interventions per symptom. The final selection of interventions was determined by open discussion and consensus-oriented voting. Each participating institution had one vote. Votes were tallied, and interventions with the highest number of votes across institutions were prioritized for inclusion. Promising but less widespread interventions were noted for future evaluation and research. **Table 2** gives an overview of the full consensus process. A targeted non-systematic literature search was conducted only for the five final best-practice interventions per symptom, aiming to support the consensus-based decisions. Sources included PubMed, the KOKON database, and national and international guidelines (e.g., AWMF, NCCN, and S3). This focused search, though not systematic in design, was intended to identify clinical evidence supporting each selected intervention. If no relevant publication was found, the field was marked as “NEI” (No Evidence Identified), indicating that no evidence was identified through this targeted non-systematic search, without implying complete absence of evidence.

**Table 2:** Consensus process (see flowchart, **Figure 1**).

1. Symptom selection: mucositis, cancer-related fatigue and nausea
2. Formation of two professional groups (physicians and nurses)
3. Collection of interventions practiced in institutions (see. **Supplementary Tables 3**-**8**)
4. Structured group sessions per symptom (4 per group, ~3–4h each)
5. Independent scoring using the standardized scoring matrix (see **Table 1**)
6. Synthesis of results
7. Two interdisciplinary consensus meetings per symptom
8. Selection of 5 best-practice interventions per symptom (see **Table 3**)
9. Targeted non-systematic literature search for the top five interventions per symptom (see **Supplementary Tables 12**-**14**)

## 3 Results

Fourteen clinical institutions from Baden-Württemberg, Germany, participated in a consensus process, oriented on a Delphi-methodology between January 2021 and December 2023. **Supplementary Table 2** summarizes the exact number of institutions and participants involved in each voting process. A total of 83 therapeutic interventions used in clinical practice for the management of CIM, CRF, and CINV were collected and documented. A summary of the results is presented in **Table 3**, with additional details available in **Supplementary Tables 9**–**11**.

**Table 3:** Practice based recommendations for CIM_CRF_CINV_consensus.

CIM results – practice based recommendation (details see in Supplementary Table 9)	Supported by literature (see Supplementary Table 12)	Level of evidence
Herbal tea sage mouthwash (Pr)	(36)	O
Ice cubes (Pr)	(6, 37–39)	Gb, SR, RCT, EC
Sea buckthorn fruit oil mouth rinses (Pr/T)	(6, 35)	EC
Frozen pineapple cubes (Pr/T)	(6)	EC
Herbal oral balm (WALA^®^ Oral Balm, containing calendula, myrrh, and ratanhia) (T) ‡		NEI

CRF results – practice based recommendation (details see in Supplementary Table 10)	Supported by literature (see Supplementary Table 13)	Level of evidence
Movement therapy (Pr/T)	(18, 21, 43)	Gb
Yarrow liver compress (T)	(33)	RCT
Viscum album therapy (T) ‡	(18, 26)	O, (Gb in QoL)
Sleep hygiene/circadian rhythm (Pr/T)	(45, 54)	SR
Hydrotherapy (T) †		NEI

CINV results - practice based recommendation (details see in Supplementary Table 11)	Supported by literature (see Supplementary Table 14)	Level of evidence
Acupressure (T)	(9, 18, 22, 23)	Gb
Aromatherapy (T)	(24)	SR
Homeopathic preparation (Nux vomica) (T)		NEI
Ginger (T)	(9)	Gb
Bitter botanicals (Gentiana) (T) †		NEI

Preventive use, T, therapeutic use; QoL, Quality of Life; Gb, Guideline-based; SR, Systematic Review; RCT, Randomized Controlled Trial; O, Observational Study; EC, Expert Consensus; NEI, No Evidence Identified (no relevant publications found in the targeted non-systematic literature search; inclusion based on clinical consensus or limited preliminary data).

†Broad category – non-standardized intervention, see Discussion.

‡Region-specific practice therapies, see Discussion.

### 3.1 Chemotherapy-induced mucositis

A total of 18 experts from 13 clinical institutions —11 physicians from 11 institutions and 7 nurses from 7 institutions — participated in the consensus process regarding the symptom of CIM. In separate group sessions, each group listed and evaluated the interventions used in their clinical routine. Physicians documented 21 interventions (**Supplementary Table 3**) and nurses 16 (**Supplementary Table 4**), resulting in a consolidated list of 26 distinct interventions after overlapping entries were harmonized. Of these, 16 interventions were included for the consolidated evaluation (see **Supplementary Table 9**): sage tea mouth rinses, ice cubes, sea buckthorn fruit oil – mouth rinses, frozen pineapple cubes, herbal oral balm (WALA Oral Balm^®^), containing calendula, myrrh, and ratanhia), oil pulling, sage and thymol mouthwash (Salviathymol^®^), herbal tea rinses with chamomile, mint, or calendula, honey-sage rinses, rosatum healing ointment (WALA^®^), myrrh tincture with local anesthetic rinse (Repha Os^®^), viscous linseed solution, calendula essence (diluted), homeopathic preparation (Traumeel^®^), anthroposophic medicinal preparation (Stibium metallicum D6), and herbal oil containing matricaria recutita and salvia officinalis (Helago^®^). Risks and contraindications were discussed and included in the recommendation process (see **Table 4**). The selection was determined by a final vote among participants. Based on this and interdisciplinary discussion, five interventions were selected as best-practice recommendations: sage tea mouth rinses, ice cubes, sea buckthorn oil mouth rinses, frozen pineapple cubes, and herbal oral balm (WALA Oral Balm^®^). Sage mouth rinses received the highest number of 9/13 votes, and the remaining four interventions each received 6/13 votes. Sage rinses, exclusively for preventive purposes, were used in nine different institutions, and scored 3/5 from both professional groups for clinical effectiveness. Ice cubes, also used preventively, were used in five institutions, with clinical effectiveness ratings of 3/5 (physicians) and 3/5 (nurses). Sea buckthorn oil rinses, applied both preventively and therapeutically, were used in three institutions and received scores of 4/5 (physicians) and 5/5 (nurses). Frozen pineapple cubes, also used for both prevention and treatment, were documented in six institutions, with scores of 4/5 (physicians) and 3/5 (nurses). Herbal oral balm (WALA Oral Balm^®^), used exclusively for therapeutic purposes, was applied in four institutions, with a clinical effectiveness score of 3/5 in both groups.

**Table 4:** Potential risks or contraindications.

CIM results –practice based recommendation	Potential risks	Contraindications
Herbal tea sage mouthwash	Thujone: • neurotoxic/convulsant • in high doses potential hepatotoxicity/nephrotoxicity with long-term or excessive use	• Pregnancy and lactation (teratogenic/neurotoxic risk) • Epilepsy or seizure disorders
Ice cubes	• Dental sensitivity • Oral mucosal trauma • Worsening of chemotherapy-induced neuropathy (e.g., oxaliplatin)	• Severe cold intolerance
Sea buckthorn fruit oil mouth rinses	• Allergic reactions • Prolonged use may cause discoloration of the teeth.	There are no known contraindications
Frozen pineapple cubes	• Allergic reactions • Mucosal irritation in severe mucositis	• Coagulation disorders or anticoagulation therapy
Herbal oral balm (WALA^®^ Oral Balm, containing calendula, myrrh, and ratanhia)	• Calendula → allergic reactions (Asteraceae family) • Myrrh → possible coagulation effects, local irritation • Ratanhia → high tannin content may cause GI irritation • Potential hepatotoxicity with prolonged use	• Allergy to Asteraceae plants or sensitivity to other ingredients • Coagulation disorders or anticoagulation therapy • long-term use not recommended

CRF results practice based recommendation	Potential risks	Contraindications
Movement therapy	• Risk of musculoskeletal strain, falls, or injury if intensity not adapted • fatigue exacerbation if overexertion	• Unstable bone metastases • Severe cardiopulmonary disease • Thrombocytopenia/neutropenia (depending on severity); exercise must be tailored
Yarrow liver compress	• Local skin irritation • Allergic reactions	• Acute abdominal inflammations • Known allergy to Asteraceae
Viscum album therapy	• Allergic reactions • Potential Drug interactions	• Acute inflammatory diseases • Active autoimmune diseases • Brain tumors/brain metastases (risk of increased edema) hematologic cancers
Sleep hygiene/circadian rhythm	No risks	No Contraindications
Hydrotherapy	• Risk of circulatory stress (hot–cold alternation) • Skin irritation • Burns if temperature control inadequate	• Severe peripheral neuropathy • Peripheral vascular disease • Cardiac instability • Open wounds/ulcerations

CINV results practice based recommendation	Potential risks	Contraindications
Acupressure	• Local pain • Skin irritation	• Avoid in inflamed or injured skin
Aromatherapy	• Allergic reactions • Bronchospasm • Mucosal irritation	• Asthma • Known allergies • Caution with open mucosal lesions
Homeopathic preparation (Nux vomica)	No risks	No Contraindications
Ginger	• acid reflux • GI upset • Possible increased bleeding risk (anticoagulant/antiplatelet therapy) • Potential Drug interactions	• Sensitivity (allergy) • Pregnancy and breastfeeding • Anticoagulant or antiplatelet therapy • Gallstones
Bitter botanicals (Gentiana) (T)	• Gastric irritation • Nausea if overdosed • High tannins	• Hypersensitivity/allergy • Peptic ulcer disease • Gastric hyperacidity

Regarding the targeted non-systematic literature search, sage mouth rinses were supported by a clinical recommendation in the S3 guideline (18) and limited individual studies with widespread traditional use. The guideline emphasizes the use of high-quality pharmaceutical-grade sage, rather than simple tea infusions. Based on evidence from randomized trials cryotherapy with ice cubes is recommended by the S3 guideline, particularly for the prevention of mucositis during short-term 5-FU chemotherapy (18). Sea buckthorn fruit oil mouth rinses were supported by positive clinical experience in several centers, with indications of very good effectiveness for both prophylactic and therapeutic use (18).

Frozen pineapple cubes were supported by clinical experience in several centers and are included as a recommended option in the current S3 guideline on supportive care, indicating good effectiveness for therapeutic use (18). No relevant literature was identified for herbal oral balm during the targeted non-systematic search; however, clinical experience strongly endorses its use. **Supplementary Table 12** presents full details of the literature findings.

In addition, three further interventions—homeopathic preparation (Traumeel^®^), anthroposophic medicinal preparation (Stibium metallicum D6) and herbal oil containing Matricaria recutita and Salvia officinalis (Helago^®^)—were not included among the top five due to limited institutional use but were retained as complementary recommendations based on strong clinical endorsement and high scores in perceived clinical effectiveness. These therapies, although evaluated by a smaller number of centers, were consistently rated ≥4 out of 5 for their clinical benefit and are suggested for further research and broader implementation.

### 3.2 Cancer-related fatigue

A total of 19 experts, representing 13 institutions: 12 physicians and 7 nursing professionals participated in the consensus process on the management of CRF. Physicians identified and evaluated 23 interventions and the nursing group 14 (see **Supplementary Tables 5**, **6**), resulting in a consolidated list of 33 distinct interventions after harmonizing overlapping entries. 13 of these distinct interventions were included for the consolidated evaluation. Risks and contraindications were discussed and included in the recommendation process. (see **Table 4**) The 13 interventions included: acupressure, movement therapy, yarrow liver compress, viscum album therapy, sleep hygiene/circadian rhythm, hydrotherapy, homeopathic preparation (phosphorus D6/D30), ginseng, full-body wash with lemon oil, foot bath with lemon oil, Qi Gong, eurythmy therapy and yoga. Subsequently, during the joint consensus sessions, the following five interventions were selected as final best-practice recommendations (s **Table 3**). Preventive and therapeutic interventions: Movement therapy scored the highest with 9/13 votes, a clinical score of 4/5 from both professional groups and was implemented by 13 of the 13 institutions. Sleep hygiene/circadian rhythm scored 7/13, was practiced in 6 institutions and scored 3 (physician) in clinical benefit. Therapeutic use: Yarrow liver compress, received 7/13 votes, was used in 7 institutions, received a clinical score of 4/5 from the nursing group and 3/5 from the physician group. Viscum album therapy, present in 10 institutions, received 7/13 votes, and was rated 3/5 by the medical group. It requires specific training and careful consideration of contraindications and interactions. Hydrotherapy, practiced in 11 institutions, received 5/13 votes, and a clinical score of 4/5 from the nursing group and 3/5 from the physician group.

The literature research is summarized in **Supplementary Table 13**. Movement therapy was supported by robust evidence documented in systematic reviews and meta-analyses, especially regarding its effectiveness in reducing CRF. An RCT with positive indications for the use of yarrow liver compresses in patients with metastatic cancer undergoing radiotherapy was found (33); its use is mainly supported by clinical experience and therapeutic tradition (18, 33). Viscum album therapy was backed by clinical studies, including controlled trials, highlighting its immunomodulatory effects and improvement of symptoms such as CRF and QoL (18). Sleep hygiene/circadian rhythm and hydrotherapy were supported by recommendations as educational and self-care interventions.

### 3.3 Chemotherapy-induced nausea and vomiting

A total of 15 experts from 11 clinical institutions participated in the consensus process on the management of CINV: 9 physicians and 6 nursing professionals. The physicians identified and evaluated 12 interventions, while the nursing group assessed 18 (see **Supplementary Tables 7**, **8**), resulting in a consolidated list of 24 distinct interventions after harmonizing overlapping entries. 7 of these distinct interventions were included for the consolidated evaluation (see **Table 3**). The 7 interventions included: acupressure, aromatherapy, homeopathic preparation (nux vomica), ginger, bitter botanicals, mind-body medicine and wormwood tea. During the final consensus session, five of these interventions were endorsed as best-practice recommendations: acupressure, aromatherapy, homeopathic preparation (nux vomica), ginger, and botanical bitter substances. Acupressure, particularly at point Pericardium 6 (PC6), was considered an effective, low-risk, and easy-to-apply intervention requiring minimal training, was reported in eight institutions and received the highest number of 7/11 votes, with clinical ratings of 3/5 (physicians) and 4/5 (nurses). Aromatherapy, primarily using essential oils like lemon or peppermint for inhalation, was reported in six institutions, with clinical ratings of 4/5 from physicians and nurses. Homeopathic preparation (nux vomica) was reported in five institutions, with clinical ratings of 3/5 (physicians) and 3/5 (nurses). Ginger was reported in seven institutions, with clinical ratings of 3/5 from both physicians and nurses. Bitter botanicals (gentiana lutea) were reported in ten institutions, with clinical ratings 3/5 (physicians) and 4/5 (nurses).

The literature research shows (see **Supplementary Table 14**): Acupressure was explicitly recommended in the S3 guideline (notably at point Pericardium 6 (PC6) for nausea management. Ginger was also supported by the guideline for nausea prevention in oncology patients. Aromatherapy showed inconsistent results in the literature, with no uniform guideline support. No relevant clinical studies were identified for Nux vomica. General references to the digestive-stimulating effect of bitter substances were found, but no direct evidence in the oncology context. Risks and contraindications were discussed and included in the recommendation process. (see **Table 4**) Despite limited scientific evidence in some cases, the five selected interventions were endorsed as best-practice recommendations due to their clinical applicability, low risk, and strong institutional experience among participating centers.

## 4 Discussion

The integration of complementary therapies into oncology care represents a transformative shift in managing cancer-related symptoms, extending beyond conventional treatment paradigms. As cancer therapies advance, maintaining and improving QoL has become as critical as enhancing survival rates (16, 18, 29). The recommendations developed within this study, grounded in clinical expertise and informed by evidence, provide a relevant pillar of evidence-based medicine (17), and offer practical and effective ways to address some of the most debilitating symptoms of cancer therapy, such as CIM, CRF, and CINV. The following sections provide an overview of the selected practice-based recommendations for each symptom, emphasizing their potential to be integrated into clinical care as practical, low-risk strategies. Rather than offering a comprehensive evaluation, this work highlights the clinical experience and feasibility of these measures as a valuable contribution to improving QoL for cancer patients and paving the way for future research to confirm these clinical observations.

It should be noted that the consensus recommendations presented in this work reflect the clinical expertise and practice traditions of integrative oncology centers located in Baden-Württemberg, Germany. While these institutions bring extensive experience in complementary and integrative care—including approaches such as traditional European and anthroposophic medicine—the panel did not include experts from other traditions such as Ayurveda or traditional Chinese herbal medicine. As such, the perspectives and practices represented here may not fully reflect the diversity of integrative oncology approaches practiced across Germany or internationally.

Mucositis remains one of the most challenging side effects of cancer treatment, affecting 40-60% of patients undergoing chemotherapy or radiation therapy (6, 34).

The consensus practice-based recommendations for managing CIM focused on interventions with the highest clinical effectiveness, ease of use, and least risk, with sage mouth rinses, ice cubes, sea buckthorn fruit oil mouth rinses, frozen pineapple cubes, and herbal oral balm (WALA Oral Balm^®^) emerging as the top recommendations. Interventions such as sea buckthorn fruit oil mouth rinses were endorsed based on strong positive experiences in a small number of institutions, although without broad implementation or high-level clinical trial evidence (35). A preliminary clinical study by Steinmann et al. (2022) also supports its potential benefits in oral mucositis. These consensus-based selections highlight promising practices that require further study before generalization.

The literature review supported these interventions, with sage mouth rinses being mentioned by KOKON as a commonly used approach (36), and cryotherapy (ice cubes) being backed by evidence for mucositis prevention, particularly during short-term 5-FU chemotherapy (37–39). It is important to note that at the time of our study, genetic predisposition to mucositis, such as DPD deficiency, was not yet widely recognized, although ice therapy showed a promising effect for mucositis prevention (40). Sea buckthorn oil and frozen pineapple cubes and herbal oral balm were included because of consistent positive reports from clinical practice, even though robust clinical trial evidence is lacking. Their inclusion therefore illustrates how consensus-based recommendations can integrate both guideline-supported interventions and practice-based approaches that require further validation. These practice-based recommendations are intended as adjuncts to guideline-based mucositis prophylaxis and management, which includes standardized oral care protocols, and pain control as outlined in the S3 Guideline for Supportive Therapy (10).CRF is one of the most prevalent and debilitating symptoms, affecting up to 70% of cancer patients (41). In the consensus process, movement-based therapies, such as yoga and Qi Gong are strongly supported by systematic reviews and meta-analyses (20, 42)., and are explicitly recommended in clinical guidelines (10, 18, 21, 43) These two approaches not only reduce physical CRF but also improve emotional well-being and QoL (20). Yoga was included in the consensus process for CRF but received fewer votes due to limited routine use in the participating institutions. This underrepresentation does not reflect the strength of the evidence. In fact, yoga is strongly recommended in several clinical guidelines, and should be more broadly implemented in integrative oncology, even if it was not prioritized in our regionally based consensus. Yarrow liver compresses were among the interventions rated with the highest perceived clinical benefit by the nursing group. Although current clinical research is limited, a randomized trial in metastatic cancer patients reported favorable trends despite no statistical significance (33). This underlines the relevance of further studies to examine their role within integrative oncology, especially considering their wide use and positive institutional experience in clinical settings. Although the inclusion of yarrow liver compresses as a best-practice recommendation was supported by clinical experience across several institutions, the current scientific evidence is limited to a small pilot trial, and further validation is necessary. The recommendation reflects experiential endorsement rather than guideline-level evidence. However, the method is highly valued in nursing practice for its perceived benefits, and this strong practical endorsement contributed to its inclusion in the consensus. Particularly the nursing group also rated hydrotherapy, in the form of partial baths or compresses, highly. Although external scientific evidence is limited, the consensus process highlighted this intervention’s potential value due to its calming, revitalizing, and regulatory effects on the nervous system based on internal evidence. These applications can be easily implemented in clinical or home settings and may be taught to patients as part of structured self-care routines. Educational materials such as instructional videos, brochures, or group classes—online or in person—could support the safe and effective use of these methods as self-help strategies to manage CRF (44). Although acupressure did not achieve the highest score required to be included in the top five best-practice recommendations, it was also discussed as a promising supportive measure. In particular, the Zick et al. study (2016) showed that self-administered acupressure significantly reduced CRF in breast cancer survivors (23). This suggests that non-invasive and low-cost approaches like acupressure may be a valuable addition to future self-management strategies for CRF, complementing more established integrative practices.

Our results highlight the perceived clinical benefit of lifestyle-based interventions, particularly those aimed at improving sleep hygiene and maintaining regular daily rhythms (45). These approaches were rated highly by both professional groups and are already implemented in several participating institutions. Given their low implementation burden and minimal risk profile, such strategies offer a promising and accessible avenue for addressing persistent CRF symptoms in oncology care. Viscum album therapy was rated highly for its clinical benefit, particularly by the physician group, and was already implemented in eight of the participating institutions. While its use requires medical supervision and specific training, a recent German review highlights its supportive role in managing CRF in breast cancer patients (26), though further rigorous trials are needed to confirm these findings in broader populations. These practice-based recommendations are intended as adjuncts to guideline-based management of CRF. Conventional supportive care emphasizes structured exercise programs, psychosocial interventions, and, when indicated, pharmacologic options. In line with this, the ASCO–SIO guideline update (2024) (21) and the NCCN Guidelines for Fatigue (Version 2.2025) (43) also highlight yoga and other mind–body therapies as evidence-based options. Our consensus therefore complements these established approaches.

CINV remains one of the most common and distressing symptoms experienced by cancer patients, particularly during chemotherapy (46). In our consensus process, acupressure and ginger stood out as the most consistently supported by both expert groups and literature findings (18) These interventions were rated highly for their applicability, minimal side effects (22), and ease of integration into standard clinical workflows. Acupressure, particularly using the Pericardium 6 (PC6) point, received recommendations from the S3 guideline on complementary medicine in oncology (S3 LL Komp), which states it “can be recommended” for nausea management. The antiemetic effect of Pericardium 6 (PC6) stimulation is plausibly explained by modulation of vagal and brainstem emetic pathways, normalization of gastric myoelectric activity, and possible interaction with serotonergic signaling (5-HT3) (47, 48).Ginger has shown modest benefits for acute CINV. A recent meta-analysis of 35 RCTs reported a reduction in severe nausea and vomiting when combined with standard antiemetic, with only mild adverse effects (<3%) (49). Proposed mechanisms include possible partial 5-HT3 antagonism and an influence on gastric motility (50). Despite its favorable safety profile, results remain heterogeneous, and the S3 Guideline concludes that current evidence is insufficient for a formal recommendation (18). Caution is advised in patients on anticoagulants due to case reports suggesting possible interactions (50).

Aromatherapy is not specifically recommended in the S3 guideline for oncology patients nor international guidelines did issue a recommendation due to inconsistent evidence. However, a recent meta-analysis of 25 randomized controlled trials (2024) reported a modest reduction in cancer-related nausea with aromatherapy, although effects on vomiting remained inconclusive and heterogeneity was high. Taken together, these findings suggest that aromatherapy may be considered as an optional adjunctive intervention, but further high-quality studies are required before broad clinical application can be recommended (24). The literature review did not identify any relevant studies supporting the efficacy of nux vomica for nausea, despite its reported use in several institutions. These results underscore the importance of ongoing research and critical evaluation before widespread clinical adoption. While aromatherapy has shown some potential for relieving nausea, particularly when combined with other complementary therapies like acupressure, the KIM-BW consensus acknowledges the need for further studies to validate its use across diverse patient populations (51). The inclusion of bitter substances such as Gentian lutea in the recommendations for CINV reflects traditional use in naturopathic medicine where they are believed to support digestive and autonomic functions. While some practitioners report positive clinical effects, robust scientific evidence in the oncology context is lacking. Therefore, this recommendation should be viewed as experience-based and interpreted with caution until further validation becomes available. Our consensus-based interventions, including acupressure, ginger, and selected experiential approaches such as aromatherapy are therefore intended to complement—not replace—these established protocols. These practice-based recommendations are intended as adjuncts to standard antiemetic prophylaxis and treatment. Conventional supportive care relies on pharmacological antiemetics tailored to the emetogenic risk of chemotherapy, as outlined in the NCCN Antiemesis Guidelines (Version 1.2025) and the MASCC/ESMO Antiemetic Guidelines (2023) (9).

While this consensus-based approach has successfully developed a set of integrative treatment recommendations, it is essential to acknowledge the study limitations. First, reliance on expert opinion, although well supported by clinical experience, introduces the potential for bias. This is particularly relevant in areas where high-quality clinical trials are lacking, and further research is needed to validate the long-term effectiveness of these therapies. While our process was oriented on a Delphi methodology to integrate diverse expert opinions, it cannot fully substitute the rigor of large-scale randomized trials (52). Nevertheless, KIM oncological treatment recommendations may offer a practical tool to increase professional awareness of these methods and generate ideas for future studies. Additionally, the irregular availability of these complementary therapies across institutions creates inequalities in the pursuit of evidence and in establishing standardized treatment approaches, which in turn hinders acceptance within the broader healthcare system. It should be noted that our consensus reflects the specific practice context of integrative oncology centers in Baden-Württemberg, Germany. Some remedies frequently used in Germany, including mistletoe preparations and products like WALA Oral Balm, are not widely available in many other healthcare systems, which limits their transferability and generalizability. Some intervention categories used in this study, such as hydrotherapy or bitter herbs, reflect traditional institutional practices rather than standardized clinical protocols. These categories may include a variety of applications (e.g., Kneipp compresses, water therapies), which differ in feasibility, safety, and available evidence. While they were included in the consensus process based on clinical relevance and local experience, the current evidence base does not yet allow for uniform recommendations across settings.

While this consensus was developed within German integrative oncology centers and refers mainly to national guidelines (e.g., S3, KOKON), several selected interventions—such as ginger for nausea, acupressure and acupuncture for CINV, pain, and fatigue, cryotherapy for mucositis, movement therapy, yoga for CRF, and mind-body approaches—are also endorsed by international guidelines, including ASCO-SIO, NCCN, and MASCC/ISOO, among others. In contrast, some region-specific practices, such as mistletoe, WALA balm, or Stibium metallicum preparations, reflect local availability and traditions which may limit their transferability to other healthcare systems.

Future studies should focus on expanding access to these therapies, particularly in resource-limited settings, and on conducting large-scale trials to confirm the clinical effectiveness of simple, low-cost, and low-risk interventions. These could eventually be established as self-help strategies that reduce symptoms and improve patients’ QoL.

The relevance of integrative oncology extends beyond clinical feasibility or expert consensus—it reflects the clearly expressed needs of patients. Studies consistently show that up to 60% of cancer patients in Germany regularly use complementary medicine, while over 80% wish to receive qualified counseling about its benefits, limitations, and risks (27). Despite widespread patient use, complementary therapies are often initiated without physician involvement, underscoring the need to better inform and integrate these practices into routine care (14). Patients seek integrative medicine for multiple reasons: to actively participate in their healing process, to mitigate side effects of conventional treatment, and to pursue a more holistic and individualized care experience (29, 53). This demand aligns with the core principles of integrative oncology, which emphasize patient-centered, evidence-informed care that incorporates natural products, mind-body practices, and lifestyle support alongside standard therapies (16).

Our consensus reflects this evolving landscape. By integrating both institutional perspectives and patients’ expectations, it aims to support informed, shared decision-making processes that enhance quality of life (QoL), patient satisfaction, and the therapeutic alliance.

## 5 Conclusion

The recommendations developed through this consensus process provide a foundation for the integration of complementary therapies into oncology care. While further research is needed to fully validate some of these therapies, the available clinical experience supports their role in alleviating patient symptoms. This multidisciplinary, patient-centered approach may enhance treatment effectiveness and aligns with the broader goals of modern oncology, which aim not only to prolong life but also to promote the overall well-being of cancer patients.

## Data Availability

The original contributions presented in the study are included in the article/**Supplementary Material**. Further inquiries can be directed to the corresponding authors.
