# Supplementary material for: Expert-guided approaches to complementary interventions for common side effects of cancer therapies: a practice-based perspective from integrative oncology centers in Baden-Württemberg, Germany
Source: Front Oncol. 2025 Nov 6;15:1667298. doi: 10.3389/fonc.2025.1667298 (PMC12631479; doi:10.3389/fonc.2025.1667298)
Supplement: Supplementary file 1 [file Table1.docx]

**Supplement 1: Summary of Professional Qualifications of Consensus Participants (Alphabetical)**

| **Name** | **Institution** | **Academic Title** | **Role** | **Specialization** | **Complementary Medicine** | **Years of Experience** |
| --- | --- | --- | --- | --- | --- | --- |
| Thomas Breitkreuz | Paracelsus-Krankenhaus Unterlengenhardt | Dr. med. | P | Internal Medicine, Palliative Medicine | Anthroposophic Medicine | 24+ yrs |
| Jürgen Brust | Diakonissen Krankenhaus Mannheim | Dr. med. | P | Hematology and Oncology |  | 20+ yrs |
| Steffi Frenzel | Kreisklinikum Heidenheim |  | N | Oncology Nursing | Anthroposophic Nursing | 15+ yrs |
| Julia Gottfried | Klinik Öschelbronn | Dr. med. | P | Hematology and Oncology, Palliative Medicine | Integrative Oncology, TEM | 15+ yrs |
| Wolfgang Heyl | Universitätsfrauenklinik der Paracelsus Medizinischen Universität, Salzburg, Austria | Prof. Dr. med | P/R/T | Gynecology | TEM | 30+ yrs |
| Stefan Hiller | Die Filderklinik | Dr. med. | P | Hematology and Oncology | Integrative Oncology / Anthroposophic Medicine | 20+ yrs |
| Ralf Hofheinz | University Medical Center Mannheim | Prof. Dr. med. | P/R/T | Hematology and Oncology | Anthroposophic Medicine | 25+ yrs |
| Meike Jocher | Klinik Öschelbronn |  | N | Oncology Nursing | Anthroposophic Nursing | 15+ yrs |
| Elke Kaschdailewitsch | Die Filderklinik |  | N | Oncology Nursing, Palliative Care | Anthroposophic Nursing | 21+ yrs |
| Klaus Kramer | University Hospital Ulm | Prof. Dr. med.  MSc | P/R/T | Oncological Surgery, Advanced Oncology (MSc) | Integrative Oncology, Lifestyle Medicine, TEM, Mind-Body-Medicine, Anthroposophic Medicine, certified Meditation Teacher (MBSR), trained MBCL-Teacher (CMF) | 30+ yrs |
| Hans Lampe | Rems-Murr-Kliniken (RMK) Winnenden | Dr. med. | P | Internal Medicine, Hematology and Oncology, Chinese Medicine | Chinese Medicine | 30+ yrs |
| Maria Livas | Städisches Krankenhaus Karlsruhe |  | P | Internal Medicine, Palliative Medicine | Anthroposophic Medicine | 16 +yrs |
| Heike Mönnich | Klinikum Esslingen | Dr. med. | P | Hematology and Oncology | Hematology and Oncology, Palliative Medicine | 15+ yrs |
| Claudia Raichle | Paul-Lechler Krankenhaus Tübingen | Dr. med. | P | Internal Medicine, Gastroenterology, Geriatrics, Palliative Medicine | Anthroposophic Medicine, TEM | 20+ yrs |
| Jane Reuter | Paul-Lechler Krankenhaus Tübingen | MSc | N | Oncology Nursing | Anthroposophic Nursing | 15+ yrs |
| Jens-Paul Seldte | RKH Krankenhaus Bietigheim-Bissingen | Dr. med. | P | Gynecological Oncology | TEM | 5 + yrs |
| Andreas Schmitt | Die Filderklinik |  | P | Visceral Surgery | Anthroposophic Medicine | 15+ yrs |
| Sigune Singer-Bayrle | Paracelsus-Krankenhaus Unterlengenhardt |  | N | Oncology Nursing | Anthroposophic Nursing | 20+ yrs |
| Jan Valentin | University Hospital Tübingen | Dr. med. | P/R | Family Medicine | Traditional Chinese Medicine | 12+ yrs |
| Theresa Wagner | Robert Bosch Krankenhaus Stuttgart | MSc | N/R | Oncology Nursing | Auricular Acupuncture | 6+ yrs |
| Annkathrin Weise | Klinikum Esslingen | BSc | N | Oncology Nursing | Anthroposophic Nursing | 12+ yrs |
| Marcela Winkler | Robert Bosch Krankenhaus Stuttgart | Dr. med. | P/R | Family Medicine / Pain Management / Palliative Medicine | TEM, Acupuncture, Mind-Body Medicine, Integrative Oncology, Medical Aromatherapy, Anthroposophic Medicine | 25+ yrs |

Abbreviations: P = Physician, N = Nurse, R= Research, T= University Teacher (Prof.), RN = Registered Nurse, MSc = Master of Science, BSc = Bachelor of Science, TEM = Traditional European Medicine
