# Supplementary material for: Expert-guided approaches to complementary interventions for common side effects of cancer therapies: a practice-based perspective from integrative oncology centers in Baden-Württemberg, Germany
Source: Front Oncol. 2025 Nov 6;15:1667298. doi: 10.3389/fonc.2025.1667298 (PMC12631479; doi:10.3389/fonc.2025.1667298)
Supplement: Supplementary file 2 [file Table2.docx]

**Supplement 2: Participating Institutions and Consensus Contributions (Alphabetical)**

| **Institution** | **Participants** | **Mucositis**  **CIM** | **Cancer- Related Fatigue CRF** | **Nausea**  **CINV** |
| --- | --- | --- | --- | --- |
| Diako Krankenhaus Mannheim, Germany | Dr.Jürgen Brust (P) | P |  |  |
| Die Filderklinik, Filderstadt, Germany | Dr. Stefan Hiller (P);  Dr. Andreas Schmitt (P);  Elke Kaschdailewitsch (N) | P – N | P – N | P – N |
| Klinik Öschelbronn, Germany | Dr. Julia Gottfried (P)  Meike Jocher (N) | P – N | N | N |
| Kreisklinikum Heidenheim, Germany | Steffi Frenzel (N) | N | N | N |
| Paracelsus-Krankenhaus Unterlengenhardt, Germany | Dr. Thomas Breitkreuz (P);  Sigune Singer-Bayrle (N) | P – N | P – N | N |
| Paul-Lechler- Krankenhaus Tübingen, Germany | Dr.Claudia Raichle (P);  Jane Reuter (N) | N | P – N |  |
| Rems-Murr Klinikum Winnenden, Germany | Dr. Hans Lampe (P) |  | P | P |
| RKH Kliniken Ludwigsburg, Germany | Prof. Dr. Wolfgang Heyl (P) | P | P | P |
| Robert Bosch Hospital, Stuttgart, Germany | Dr.Marcela Winkler (P);  Theresa Wagner (N) | P – N | P – N | P – N |
| Klinikum Esslingen, Esslingen, Germany | Dr. Heike Mönnich (P);  Annkathrin Weise (N) | N | P – N | P – N |
| Städtisches Krankenhaus Karlsruhe, Germany | Maria Livas (P) | P | P | P |
| Department of General and Visceral Surgery, Section Integrative Medicine, University Hospital Ulm, Germany | Prof. Dr. Klaus Kramer (P) | P | P | P |
| University Medical Center Mannheim, Germany | Prof. Dr. Ralf Hofheinz (P) | P | P |  |
| RKH Krankenhaus Bietigheim-Bissingen, Germany | Jens-Paul Seldte (P) | P | P | P |
| Total physicians and nurses |  | 11 P- 7 N | 12 P- 7 N | 9 P- 6 N |
| Total institutions |  | 13 | 13 | 11 |

Abbreviations: P = Physician, N = Nurse

**Summary:** 14 institutions participated in the consensus process, with 14 physicians and 7 nurses. Additionally, one expert (Dr. Jan Valentini- Institute of General Practice and Interprofessional Care, University Hospital Tübingen, Germany) contributed to the literature research.
