# Supplementary material for: Expert-guided approaches to complementary interventions for common side effects of cancer therapies: a practice-based perspective from integrative oncology centers in Baden-Württemberg, Germany
Source: Front Oncol. 2025 Nov 6;15:1667298. doi: 10.3389/fonc.2025.1667298 (PMC12631479; doi:10.3389/fonc.2025.1667298)
Supplement: Supplementary file 3 [file Table3.docx]

**Supplement 3: Chemotherapy-Induced-Mucositis_(CIM)_Interventions_Physicians**

| **Intervention** | **Special Notes** | **Inter-actions** | **Contra-indications** | **Required Training** | **Feasi-bility** | **Time Effort** | **Institutional Use (n/total)** | **Effective-ness** |
| --- | --- | --- | --- | --- | --- | --- | --- | --- |
| Calendula essence (diluted) | Pr |  | Allergies | 1 | 1 | 1 | PU/LB/ BB /RB= 4/11 | 3 |
| Frozen pineapple cubes | Pr / T |  | Allergies | 1 | 2 | 2 | LB /BB/ M = 3/11 | 4 |
| Healing earth mouthwash | Pr |  | Stem cell transplantation | 1 | 1 | 1 | RB= 1/11 | 2 |
| Herbal chamomile mouthwash | Pr |  |  | 1 | 1 | 1 | KA/UK/LB/ = 3/11 | 3 |
| Sage mouth rinses | Pr / T |  | Allergies | 1 | 1 | 1 | KA /UK/DK/LB/BB = 5/11 | 3 |
| Herbal oral balm (WALA Oral Balm ®), containing calendula, myrrh, and ratanhia) | T |  | Open lesions | 1 | 1 | 1 | UK/F/PU = 3/11 | 3 |
| Honey and sage rinse | T  N: For radiation-induced esophagitis |  |  | 1 | 1 | 1 | F/RB/PU = 3/11 | 3 |
| Ice cubes | Pr |  |  | 1 | 1 | 1 | RB/LB /BB = 3/11 | 3 |
| Linseed mucilage | Pr |  |  | 1 | 1 | 1 | UK/DK = 2/11 | 2 |
| Mare’s milk | T |  |  | 1 | 1 | 1 | F= 1/11 | 3 |
| Myrrh tincture rinse (e.g., Repha Os®) | T  N: For targeted local application in oral aphthae |  | Allergies | 1 | 2 | 1 | LB/BB/KA/DK = 4/11 | 3 |
| Oil pulling | Pr / T |  |  | 1 | 1 | 1 | KA/PU/DK = 3/11 | 3 |
| Patient education: prevention of mechanical irritation | Pr |  |  | 1 | 1 | 1 | DK/F/RB = 3/11 | 2 |
| Ratanhia compound mouthwash | T |  |  | 1 | 1 | 1 | UK/M/F/DK = 4/11 | 3 |
| Rosatum-based healing ointment (WALA®) | Pr / T |  |  | 1 | 1 | 1 | KA/P/Ö= 3/11 | 3 |
| Sage and thymol mouthwash (Salviathymol®) | Pr / T |  | Open lesions | 1 | 1 | 1 | KA/UK/PU/DK= 4/11 | 2 |
| Sea buckthorn fruit oil rinses | Pr / T |  |  | 1 | 1 | 1 | KA/PU/RB= 3/11 | 4 |
| Sodium selenite | Pr / T  N: Head and neck cancers stem cell transplantation. |  |  | 1 | 1 | 1 | RB= 1/11 | 3 |
| Anthroposophic medicinal preparation (Stibium metallicum D6) | Pr / T |  |  | 1 | 1 | 1 | F=1/11 | 4 |
| Homeopathic preparation (Traumeel®) | Pr /T |  |  | 1 | 1 | 1 | RB =1/11 | 4 |
| Zinc supplementation | Pr / T  N: Radiation-induced mucositis |  |  | 1 | 1 | 1 | RB= 1/11 | 3 |

Abbreviations: BB: RKH Krankenhaus Bietigheim-Bissingen, Germany; DK: Diako Krankenhaus Mannheim, Germany; F: Die Filderklinik, Filderstadt, Germany; KA: Städtisches Krankenhaus Karlsruhe, Germany; LB: RKH Kliniken Ludwigsburg, Germany; M: University Medical Center Mannheim, Germany; Ö: Klinik Öschelbronn, Germany; P: Paul-Lechler- Krankenhaus Tübingen, Germany; PU: Paracelsus-Krankenhaus Unterlengenhardt, Germany; RB: Robert Bosch Hospital, Stuttgart, Germany; UK: Department of General and Visceral Surgery, Section Integrative Medicine, University Hospital Ulm, Germany;

Institutional Use (n/total): Number of institutions applying the intervention / total number of participating institutions (11)

Pr: preventive use, T: therapeutic use; N: Note.
