# Supplementary material for: Expert-guided approaches to complementary interventions for common side effects of cancer therapies: a practice-based perspective from integrative oncology centers in Baden-Württemberg, Germany
Source: Front Oncol. 2025 Nov 6;15:1667298. doi: 10.3389/fonc.2025.1667298 (PMC12631479; doi:10.3389/fonc.2025.1667298)
Supplement: Supplementary file 4 [file Table4.docx]

**Supplement 4: Chemotherapy-Induced_Mucositis_(CIM)_Interventions_Nurses**

| **Intervention** | **Special Notes** | **Inter-actions** | **Contra-indications** | **Required Training** | **Feasi-bility** | **Time Effort** | **Institutional Use (n/total)** | **Effective-ness** |
| --- | --- | --- | --- | --- | --- | --- | --- | --- |
| Calendula blossom tea | Pr |  |  | 2 | 4 | 2 | F=1/8 | 2 |
| Frozen pineapple cubes | Pr / T |  |  | 1 | 4 | 1 | KA/UK/F= 3/8 | 3 |
| Herbal oil (Helago®), containing matricaria recutita and salvia officinalis) | T |  |  | 1 | 4 | 1 | Ö/F= 2/8 | 4 |
| Herbal oral balm (WALA Oral Balm®), containing calendula, myrrh, and ratanhia | T |  |  | 1 | 3 | 1 | PU/KA/F= 3/8 | 3 |
| Herbal chamomile tea mouthwash | N: anti-inflammatory |  |  | 2 | 4 | 3 | RB= 1/8 | 3 |
| Herbal equisetum tea mouthwash |  |  |  | 2 | 4 | 2 | KA= 1/8 | 3 |
| Sage mouth rinses | Pr, N: antiseptic. effect |  |  | 2 | 4 | 3 | ES/KA/Ö/F/HH= 5/8 | 3 |
| Herbal thymol tea mouthwash | N: antibacterial |  |  | 2 | 4 | 2 | Ö= 1/8 | 3 |
| Ice cubes | Pr , N: pain-relieving |  |  | 1 | 3 | 1 | ES/RB/Ö= 3/8 | 3 |
| Linseed mucilage | Pr |  |  | 2 | 4 | 2 | F= 1/8 | 3 |
| Mare's milk | T |  | allergy, immune suppression, infection risk | 1 | 4 | 1 | F= 1/8 | 4 |
| Marshmallow root tea rinse | Pr / T |  |  | 2 | 4 | 2 | F= 1/8 | 3 |
| Myrrh tincture rinse (e.g., Repha Os®) | T |  |  | 1 | 4 | 1 | KA= 1/8 | 4 |
| Sea buckthorn fruit oil mouth rinse | Pr / T |  |  | 1 | 4 | 1 | KA/RB= 2/8 | 5 |
| Anthroposophic medicinal preparation (Stibium metallicum D6) | Pr / T |  |  | 1 | 4 | 1 | F= 1/8 | 4 |
| Sunflower oil and a drop of lemon | Pr, N: for oil pulling |  |  | 2 | 4 | 2 | F/KA= 2/8 | 2 |

**Abbreviations:** ES: Klinikum Esslingen, Esslingen, Germany; F: Die Filderklinik, Filderstadt, Germany; HH: Kreisklinikum Heidenheim, Germany; KA: Städtisches Krankenhaus Karlsruhe, Germany; Ö: Klinik Öschelbronn, Germany; PU: Paracelsus-Krankenhaus Unterlengenhardt, Germany; RB: Robert Bosch Hospital, Stuttgart, Germany; UK: Department of General and Visceral Surgery, Section Integrative Medicine, University Hospital Ulm, Germany

**Institutional Use** (n/total): Number of institutions applying the intervention / total number of participating institutions (8) Pr: preventive use, T: therapeutic use; N: Notice
