# Supplementary material for: Expert-guided approaches to complementary interventions for common side effects of cancer therapies: a practice-based perspective from integrative oncology centers in Baden-Württemberg, Germany
Source: Front Oncol. 2025 Nov 6;15:1667298. doi: 10.3389/fonc.2025.1667298 (PMC12631479; doi:10.3389/fonc.2025.1667298)
Supplement: Supplementary file 5 [file Table5.docx]

**Supplement 5: Cancer-Related-Fatigue_(CRF)_Interventions_Physicians**

| **Intervention** | **Special Notes** | **Interactions** | **Contraindications** | **Required Training** | **Feasi-bility** | **Time Effort** | **Institutional Use (n/total)** | **Effectiv-ness** |
| --- | --- | --- | --- | --- | --- | --- | --- | --- |
| Acupressure | N: 30 min daily. T |  |  | 2 | 3 | 4 | RM/RB= 2/12 | 3 |
| Acupuncture | T |  | Thrombocytopenia, neutropenia, needle phobia, fever or signs of systemic infection, severe skin conditions at needle sites, history of uncontrolled epilepsy, implanted pacemaker (for electroacupuncture), unstable cardiovascular conditions, pregnancy | 5 | 5 | 4 | RB/LB/ BB/RM= 4/12 | 3 |
| Cardiodoron ® (anthroposophic medicinal product) | T |  |  | 3 | 3 | 1 |  | 3 |
| Decoctions of Chinese Medicine | N: Requires Chinese medical diagnosis. T | Potential interactions should be carefully evaluated. |  | 5 | 5 | 4 | RM/LB = 2/12 | 3 |
| Eurythmy therapy | Pr/T |  |  | 5 | 3 | 4 | PU/HH/Ö/F/UK= 5/12 | 3 |
| Fasting | Pr/T |  | Unstable diabetes mellitus, underweight or eating disorders, pregnancy or breastfeeding, severe heart or kidney disease, active infections, recent surgery, uncontrolled hyperthyroidism, psychiatric disorders (e.g. severe depression), and frailty or advanced age with risk of malnutrition. | 3 | 5 | 4 | LB/BB/RB= 3/12 | 3 |
| Ginseng | N: 1000–2000 mg daily for at least 8 weeks before clinical effects can be evaluated. /T | Potential interactions should be carefully evaluated. | uncontrolled hypertension, insomnia, hormone-sensitive conditions (such as breast, uterine, or ovarian cancer), pregnancy and breastfeeding, bleeding disorders or concurrent use of anticoagulants, autoimmune diseases | 3 | 3 | 1 | F/RB/KA= 3/12 | 3 |
| Helleborus niger D3- D6 (homeopathic preparation) | T |  |  | 3 | 3 | 1 | PU/KA/UK/HH/M/Ö= 6/12 | 3 |
| Hydrotherapy | T  N:In the morning/Pr-T |  |  | 2 | 2 | 1 | LB/BB/HH/RB/UK/PU//F= 7/12 | 3 |
| Homeopathic preparation (Levico®) | T | Fatigue with a picture of exhaustion depression |  | 3 | 3 | 1 | F/ PU/ Ö/HH/UK/RB= 6/12 | 2 |
| Mindfulness-Based Stress Reduction | Pr/T |  |  | 5 | 4 | 4 | KA/LB/UK/M/F/RB= 6/12 | 3 |
| Movement therapy | N: Guidance. Pr/T |  | Bleeding, thrombocytopenia below 10,000/nl; caution at 10,000–20,000/nl, anemia (hemoglobin below 8 g/dl), on the day of chemotherapy with cardiotoxic or nephrotoxic agents, fever, or active infection. | 2 | 2 | 2 | RB/RM/KA/Ö/LB/UK/M/ P/BB/PU/Ö= 11/12 | 4 |
| Nutrition (advice) | Pr/T |  |  | 5 | 4 | 3 | RM/KA/Ö/RB= 4/12 | 3 |
| Homeopathic preparation (Phosphorus D6/D30) | T  N: "Phosphorus swing" morning D6 / evening D30. Day-night rhythm. |  |  | 3 | 3 | 1 | Ö/PU/HH= 3/12 | 3 |
| Power broth (TCM) | T  N: Elaborate. |  |  | 1 | 1 | 1 | RB= 1/12 | 3 |
| Qi-Gong | Pr/T |  | Risk of fracture; risk of bleeding (thrombocytes < 20,000/μl). | 5 | 5 | 4 | RB/RM/KA/LB/BB= 5/12 | 3 |
| Standardized Rhodiola rosea extract (Rhodiola rhodioLoges ®) | T |  |  | 3 | 3 | 1 | LB= 1/12 | 3 |
| Rosemary oil (aromatherapy) | T  N: in the morning. |  |  | 2 | 2 | 1 | RB= 1/12 | 3 |
| Sleep hygiene / circardian rhythm / | Pr/T |  |  | 1 | 1 | 3 | F/LB/HH/UK/KA/PU= 6/12 | 3 |
| Viscum album therapy i.v. (mistletoe) | T  N: Intervention for severe acute exacerbated fatigue in inpatient settings, administered immediately prior to chemotherapy. | Potential interactions should be carefully evaluated. | acute infections or fever; uncontrolled cardiovascular conditions; seizure disorders; active autoimmune diseases. | 4 | 4 | 2 | P/F/HH/Ö= 4/12 | 3 |
| Viscum album therapy (mistletoe) subcutaneously | T  N: 2 - 3 weekly. | Potential interactions should be carefully evaluated. | Acute inflammatory or febrile illness; autoimmune diseases in active phase; severe cardiovascular conditions (e.g., uncontrolled hypertension); active hyperthyroidism. | 4 | 3 | 1 | RB/PU/LB/BB/F/HH/KA/Ö/M/UK= 10/12 | 3 |
| Yarrow liver compress | T |  |  | 2 | 2 | 2 | RK/UK= 2/12 | 3 |
| Mindfulness-Based Stress Reduction | Pr/T |  | Risk of fracture; risk of bleeding (thrombocytes < 20,000/μl). | 5 | 5 | 4 | RB/LB/ BB= 3/12 | 3 |

Abbreviations: BB: RKH Krankenhaus Bietigheim-Bissingen, Germany; F: Die Filderklinik, Filderstadt, Germany; HH: Kreisklinikum Heidenheim, Germany; KA: Städtisches Krankenhaus Karlsruhe, Germany; LB: RKH Kliniken Ludwigsburg, Germany; M: University Medical Center Mannheim, Germany; Ö: Klinik Öschelbronn, Germany; P: Paul-Lechler- Krankenhaus Tübingen, Germany; PU: Paracelsus-Krankenhaus Unterlengenhardt, Germany; RB: Robert Bosch Hospital, Stuttgart, Germany; RM: Rems-Murr Klinikum Winnenden, Germany; UK: Department of General and Visceral Surgery, Section Integrative Medicine, University Hospital Ulm, Germany

Institutional Use (n/total): Number of institutions applying the intervention / total number of participating institutions (12)

Pr: preventive use, T: therapeutic use; N= Note
