# Supplementary material for: Expert-guided approaches to complementary interventions for common side effects of cancer therapies: a practice-based perspective from integrative oncology centers in Baden-Württemberg, Germany
Source: Front Oncol. 2025 Nov 6;15:1667298. doi: 10.3389/fonc.2025.1667298 (PMC12631479; doi:10.3389/fonc.2025.1667298)
Supplement: Supplementary file 6 [file Table6.docx]

**Supplement 6: Cancer-Related-Fatigue_(CRF)_Interventions_Nurses**

| **Intervention** | **Special Notes** | **Interactions** | **Contraindications** | **Required Training** | **Feasi-bility** | **Time Effort** | **Institutional** | **Effective-ness** |
| --- | --- | --- | --- | --- | --- | --- | --- | --- |
| Abdominal application with Cuprum oil 0.4% or ointment Cuprum met. 0.4% | T  N: relaxing (veg Neven system). |  |  | 3 | 4 | 5 | Ö= 1/6 | 3 |
| Arm rub rhythm with Prunus oil or Mallow | T  N: invigorating, harmonizing. |  |  | 5 | 4 | 5 | Ö= 1/6 | 3 |
| Coffee kidneys wrap | T |  |  | 3 | 4 | 5 | HH = 1/6 | 3 |
| Cuprum oil 0.4%- Rosemary oil | T  N: in the morning. |  |  | 4 | 4 | 5 | Ö/HH= 2/6 | 3 |
| Foot bath with lemon oil | T |  |  | 2 | 4 | 4 | ES= 1/6 | 4 |
| Foot baths with rosemary oil | T  N: rhythmization, in the morning. |  |  | 2 | 4 | 4 | Ö/F/KA/RB= 4/6 | 3 |
| Full body wash with lemon oil | T |  |  | 1 | 4 | 3 | F/HH/Ö/KA= 4/6 | 3 |
| Ginger kidneys wrap | T |  |  | 3 | 4 | 5 | HH= 1/6 | 3 |
| Hydrotherapy | Pr / T  N: in the morning. |  |  | 3 | 4 | 3 | RB/Ö/F/HH/ES/KA= 6/6 | 4 |
| Movement therapy | Pr / T |  |  | 1 | 1 | 2 | HH/KA/F/ES/RB =5/6 | 4 |
| Rosemary salt rubbing | T |  |  | 2 | 4 | 3 | HH= 1/6 | 3 |
| Yarrow liver compress | T |  |  | 4 | 3 | 5 | Ö/HH/ES/F/RB= 5/6 | 4 |
| Yarrow oil overlay10 % on liver | T |  |  | 3 | 4 | 5 | KA/Ö= 2/6 | 3 |
| Yarrow wax on liver | T |  |  | 3 | 4 | 5 | RB= 1/6 | 4 |

Abbreviations: ES: Klinikum Esslingen, Esslingen, Germany; F: Die Filderklinik, Filderstadt, Germany; HH: Kreisklinikum Heidenheim, Germany; Ö: Klinik Öschelbronn, Germany; PU: Paracelsus-Krankenhaus Unterlengenhardt, Germany; RB: Robert Bosch Hospital, Stuttgart, Germany

Institutional Use (n/total): Number of institutions applying the intervention / total number of participating institutions (6)
