# Supplementary material for: Expert-guided approaches to complementary interventions for common side effects of cancer therapies: a practice-based perspective from integrative oncology centers in Baden-Württemberg, Germany
Source: Front Oncol. 2025 Nov 6;15:1667298. doi: 10.3389/fonc.2025.1667298 (PMC12631479; doi:10.3389/fonc.2025.1667298)
Supplement: Supplementary file 7 [file Table7.docx]

**Supplement 7: Chemotherapy-Induced-Nausea and Vomiting_(CINV)_Interventions Physicians**

| **Intervention** | **Special Notes** | **Interactions** | **Contraindications** | **Required Training** | **Feasi-bility** | **Time Effort** | **Institutional Use (n/total)** | **Effective-ness** |
| --- | --- | --- | --- | --- | --- | --- | --- | --- |
| Acupuncture | T |  |  | 5 | 5 | 3 | RB/LB/BB/RM= 4/10 | 3 |
| Acupressure |  | Depressants E.g. morphine, lorazepam | asthma, allergies | 2 | 1 | 1 | RB/F/PU/KA/RM/LB/BB= 7/10 | 3 |
| Aromatherapy | T  N: Aroma stick. (Lemon-Ginger) |  |  | 3 | 1 | 1 | UK/Ö/ES/KA/RB/F= 6/10 | 4 |
| Bitter botanicals(Gentiana) | T | Content Alcohol 50% |  | 1 | 1 | 1 | HH/F/P/PU/KA/RB/Ö/LB/BB/UK= 10/10 | 3 |
| Bryophyllum | T  N: wit anxiety anticipatory nausea. |  |  | 1 | 1 | 1 | RB/F/HH/PU/Ö/P/KA/UK= 8/10 | 3 |
| Cannabinoids: dronabinol, nabilone | T  N: refractory to therapy | Numerous interactions via CYP450 should be reviewed | Psychoses, depression, bipolar, panic attacks, epilepsy | 4 | 2. | 1 | P/BB/LB/F/ES= 5/10 | 2 |
| Decoction TCM  (individualized mixtures) | T  N: Individualized therapy. | Numerous interactions should be reviewed. |  | 5 | 5 | 3 | LB/RM= 2/10 | 3 |
| Fasting / Intermittent fasting | Pr | BMI<21  Eating disorder | Diabetes mellitus | 3 | 2 | 3 | LB/BB/RB= 3/10 | 3 |
| Ginger | Pr / T  N: Tea or capsules | Aprepitant and cyclosporine, NSAIDs and insulin | Perioperative (bleeding), cholecystolithiasis | 3 | 3 | 1 | RB/F/PU/KA/RM/LB/BB= 7/10 | 3 |
| Mind-body medicine | Pr / T |  |  | 5 | 2 | 4 | RB/BB/LB= 3/10 | 2 |
| Nux vomica comp. (homeopathic preparation) | T |  |  | 1 | 1 | 1 | HH/F/LB/BB/Ö= 5/10 | 3 |
| Progressive Muscle relaxation according to Jacobson | Pr/T  Mainly anticipatory Nausea. | Aortic Aneurysm |  | 4 | 2 | 2 | RB= 1/10 | 4 |

Abbreviations: BB: RKH Krankenhaus Bietigheim-Bissingen, Germany; ES: Klinikum Esslingen, Esslingen, Germany; F: Die Filderklinik, Filderstadt, Germany; KA: Städtisches Krankenhaus Karlsruhe, Germany; LB: RKH Kliniken Ludwigsburg, Germany; Ö: Klinik Öschelbronn, Germany; PU: Paracelsus-Krankenhaus Unterlengenhardt, Germany; RB: Robert Bosch Hospital, Stuttgart, Germany; RM: Rems-Murr Klinikum Winnenden, Germany; UK: Department of General and Visceral Surgery, Section Integrative Medicine, University Hospital Ulm, Germany;

Pr: Preventive use, T: therapeutic use; N: Notice, BMI: Body Mass Index, NSAIDs: Non-Steroidal Anti-Inflammatory Drugs.

Institutional Use (n/total): Number of institutions applying the intervention / total number of participating institutions (10)
