# Supplementary material for: Expert-guided approaches to complementary interventions for common side effects of cancer therapies: a practice-based perspective from integrative oncology centers in Baden-Württemberg, Germany
Source: Front Oncol. 2025 Nov 6;15:1667298. doi: 10.3389/fonc.2025.1667298 (PMC12631479; doi:10.3389/fonc.2025.1667298)
Supplement: Supplementary file 8 [file Table8.docx]

**Supplement 8: Chemotherapy-Induced-Nausea and Vomiting_(CINV)_Interventions_Nurses**

| **Intervention** | **Special Notes** | **Inter-actions** | **Contraindications** | **Required Training** | **Feasi-bility** | **Time Effort** | **Institutional Use (n/total)** | **Effective-ness** |
| --- | --- | --- | --- | --- | --- | --- | --- | --- |
| Acupressure | T |  |  | 1 | 1 | 1 | RB/F/KA/UK= 4/7 | 4 |
| Aromatherapy | T  N: lemon |  | Allergies | 1 | 1 | 1 | KA/RB/ES= 3/7 | 4 |
| Bitter botanicals (Gentiana) | T |  |  | 1 | 1 | 1 | UK= 1/8 | 4 |
| Breathing technique | T  N: effective for a short |  |  | 1 | 1 | 1 | KA/F/UK/Ö/ES/RB/HH)= 7/7 | 2 |
| Chamomile oil overlay | Pr/ T  N: relaxing, antispasmodic |  |  | 4 | 3 | 5 | UK/Ö/F/ES/HH)/=5/8 | 3 |
| Chamomile wax | T |  |  | 3 | 4 | 5 | RB= 1/7 | 2 |
| Draining leg swab | Pr/T  N: can also be applied over lothing |  | Skin lesions | 5 | 2 | 4 | F/Ö/KU/RB/HH/KA= 6/7 | 2 |
| Ferrum ointment application | T  N: on the liver/gall bladder |  | Fiever, Cholangitis | 3 | 3 | 5 | Ö= 1/7 | 3 |
| Foot embrocation (incorporating rhythm techniques, reflexology zones) | T |  | Skin lesions | 5 | 3 | 5 | Ö/UK/F/KA/RB= 5/7 | 4 |
| Ginger | T |  | NSAIDS, Anticoagulants; Tacrolimus | 1 | 4 | 1 | RB/F/KA= 3/7 | 3 |
| Lemon juice orally | Pr/T |  | Mucositis | 1 | 1 | 1 | F= 1/7 | 2 |
| Melissa wrap | T  N: heating through |  |  | 4 | 3 | 5 | Ö= 1/7 | 3 |
| Nux vomica (homeopathic preparation) | T |  |  | 2 | 1 | 1 | Ö/HH= 2/7 | 3 |
| Oxalis essence | T  N: rhythm. Abdominal rub | Severely weakness |  | 4 | 3 | 5 | Ö/HH/UK= 3/7 | 3 |
| Wormwood tea | T |  |  | 1 | 1 | 1 | HH/KA= 2/7 | 4 |
| Yarrow tea liver compress | T  N: apply until 3 p.m. or at night as a sleep aid |  | Cholangitis, Ileus symptoms, Liver capsule pain, fever | 4 | 3 | 5 | UK/Ö/ES/HH/KA/F = 6/7 | 4 |
| Yarrow wax plant compress | T  N: apply until 3 p.m. or at night as a sleep aid |  | Cholangitis, Ileus symptoms, Liver capsule pain, fever | 3 | 3 | 5 | RB= 1/7 | 4 |
| Yarrow oil liver compress | T  N: apply until 3 p.m. or at night as a sleep aid |  | Cholangitis, Ileus symptoms, Liver capsule pain, fever | 4 | 4 | 5 | Ö/KA/HH= 3/7 | 3 |

Abbreviations: ES: Klinikum Esslingen, Esslingen, Germany; F: Die Filderklinik, Filderstadt, Germany; HH: Kreisklinikum Heidenheim, Germany; KA: Städtisches Krankenhaus Karlsruhe, Germany; Ö: Klinik Öschelbronn, Germany; RB: Robert Bosch Hospital, Stuttgart, Germany; UK: Department of General and Visceral Surgery, Section Integrative Medicine, University Hospital Ulm, Germany

Pr: preventive use, T: therapeutic use; N= Notice.

Institutional Use (n/total): Number of institutions applying the intervention / total number of participating institutions (7)
