# Supplementary material for: Expert-guided approaches to complementary interventions for common side effects of cancer therapies: a practice-based perspective from integrative oncology centers in Baden-Württemberg, Germany
Source: Front Oncol. 2025 Nov 6;15:1667298. doi: 10.3389/fonc.2025.1667298 (PMC12631479; doi:10.3389/fonc.2025.1667298)
Supplement: Supplementary file 9 [file Table9.docx]

**Supplement 9: Practice Based Recommendation for Chemotherapy-Induced Mucositis**

**Voting: Practice based recommendations for Complementary and Integrative Medicine (CIM) Consensus**

Total physicians and nurses: 10 P- 7 N

Total institutions: 13

Note to the Table: The "Number of Institutions" and "Effectiveness" columns represent the initial evaluations, as shown in Supplement 3 for physicians and Supplement 4 for nurses. The "Voting X / 13" column shows the final voting results, with each institution casting one vote.

Results - Best Practice Recommendation: (A)-(B)-(C)-(D)-(E)

| **Intervention** | **Physicians** | | **Nurses** | |  |
| --- | --- | --- | --- | --- | --- |
|  | Number of  Institutions | Effective-ness | Number of  Institutions | Effective-ness | Voting  X / 13 |
| **Preventive use** |  |  |  |  |  |
| Ice cubes (B) | 3 (RB/LB/BB) | 3 | 3 (ES/RB/Ö) | 3 | **6 /13** |
| Linseed mucilage | 2 (UK/DK) | 2 | 1 (F) | 3 | 2 /13 |
| Calendula essence (diluted) | 4 (PU/LB/BB/RB) | 3 | * | * | 1 /13 |
| Sage mouth rinses (A) | 5 (KA/UK/DK/LB/BB) | 3 | 5 (ES/KA/Ö/F/HH) | 3 | **9** /13 |
| **Therapeutic use** |  |  |  |  |  |
| Herbal oral balm (WALA Oral Balm ®) containing calendula, myrrh, and ratanhia) (E) | 3 (UK/F/PU) | 3 | 3 (PU/KA/F) | 3 | **6** /13 |
| Ratanhia compound mouthwash | 4 (UK/M/F/DK) | 3 | * | * | 4 /13 |
| **Preventive and Therapeutic use** |  |  |  |  |  |
| Frozen pineapple cubes (D) | 3 (LB/BB/M) | **4** | 3 (KA/UK/F) | 3 | **6** /13 |
| Sea buckthorn fruit oil rinses (C) | 3 (KA/PU/RB) | **4** | 2 (KA/RB) | **5** | **6** /13 |
| Rosatum-based healing ointment (WALA®) | 3 (KA/P/Ö) | 3 | * | * | 3 /13 |
| Herbal tea Chamomile - mouth rinses | 3 (KA/UK/LB) | 3 | 1 (RB) | 3 | 4 /13 |
| Oil pulling | 3 (KA/PU/DK) | 3 | * | * | 3 /13 |
| Herbal oil, containing Matricaria recutita and Salvia officinalis (Helago ®)** | * | * | 2 (Ö/F) | **4** | 2 /13 |
| Myrrh tincture rinse (e.g., Repha Os®) | 4 (LB/BB/KA/DK) | 3 | 1 (KA) | 3 | 1 /13 |
| Sage and thymol mouthwash (Salviathymol ®) | 4 (KA/UK/PU/DK) | 2 | * | * | 1 /13 |
| Anthroposophic medicinal preparation (Stibium metallicum D6)** | 1(F) | **4** | 1(F) | **4** | 1 /13 |
| Homeopathic preparation (Traumeel ®)** | 1 (RB) | **4** | * | * | 1 /13 |

* Not evaluated in the group

** **Noteworthy:** Particularly good effectiveness effects were observed in individual institutions. Special procedures should be included in the list of best practice recommendations:

**Abbreviations:** BB: RKH Krankenhaus Bietigheim-Bissingen, Germany; DK: Diako Krankenhaus Mannheim, Germany; ES: Klinikum Esslingen, Esslingen, Germany; F: Die Filderklinik, Filderstadt, Germany; HH: Kreisklinikum Heidenheim, Germany; KA: Städtisches Krankenhaus Karlsruhe, Germany; LB: RKH Kliniken Ludwigsburg, Germany; M: University Medical Center Mannheim, Germany; Ö: Klinik Öschelbronn, Germany; P: Paul-Lechler- Krankenhaus Tübingen, Germany; PU: Paracelsus-KrankenhausUnterlengenhardt, Germany; RB: Robert Bosch Hospital, Stuttgart, Germany; RM: Rems-Murr Klinikum Winnenden, Germany; UK: Department of General and Visceral Surgery, Section Integrative Medicine, University Hospital Ulm, Germany
