# Supplementary material for: Expert-guided approaches to complementary interventions for common side effects of cancer therapies: a practice-based perspective from integrative oncology centers in Baden-Württemberg, Germany
Source: Front Oncol. 2025 Nov 6;15:1667298. doi: 10.3389/fonc.2025.1667298 (PMC12631479; doi:10.3389/fonc.2025.1667298)
Supplement: Supplementary file 10 [file Table10.docx]

**Supplement 10: Practice Based Recommendation for Cancer-Related Fatigue (CRF)**

**Voting: Practice based recommendations for Consensus**

Total physicians and nurses: 11 P- 7 N - Total institutions: 13

Note to the Table: The "Number of Institutions" and "Effectiveness" columns represent the initial evaluations, as shown in Supplement 5 for physicians and Supplement 6 for nurses. The "Voting X / 13" column shows the final voting results, with each institution casting one vote.

Results - Best Practice Recommendation: (A)-(B)-(C)-(D)-(E)

| **Intervention** | **Physicians** | | **Nurses** | |  |
| --- | --- | --- | --- | --- | --- |
|  | Number of  Institution | Effective-ness | Number of  Institution | Effective-ness | Voting |
| **Therapeutic use** |  |  |  |  |  |
| Yarrow liver compress (B) | 2 (P/UK) | 3 | 5 (Ö/HH/ES/F/RB) | 4 | **7 /13** |
| Viscum album therapy (C) | **10 (**RB/PU/LB/BB/F/HH/KA/Ö/M/UK) | 3 | * | * | **7/13** |
| Hydrotherapy (E) | 7 (LB/BB/HH/RB/UK/PU/F) | 3 | 6 (RB/Ö/F/HH/ES/KA) | 4 | **5 /13** |
| Homeopathic preparation (Phosphorus D6/D30) | 3 (Ö/PU/HH) | 3 | * | * | 3 /13 |
| Ginseng | 3 (F/RB/KA) | 3 | * | * | 3 /13 |
| Full body wash with lemon oil | * | * | 4 (F/H/Ö/KA) | 3 | 4 /13 |
| Foot bath with lemon oil | * | * | 1 (ES) | 4 | 2 /13 |
| Acupressure | 7 (LB/BB/HH/RB/UK/PU/F) | 3 | * | * | 4 **/**13 |
| **Preventive and**  **Therapeutic use** |  |  |  |  |  |
| Movement therapy (A) | **12** (RB/F/RM/UK/KA/M/P/HH/LB  /BB/PU/Ö) | 4 | 5 (HH/KA/F/ES/RB) | 4 | **9 /13** |
| Sleep hygiene/circadian rhythm (D) | 6 (F/LB/HH/UK/KA/PU) | 3 | * | * | **7 /13** |
| Qi-Gong | 5 (RB/RM/KA/LB/ BB) | 3 | * | * | 5 /13 |
| Eurythmy therapy | 5 (PU/HH/Ö/F/UK) | 3 | * | * | 4 /13 |
| Yoga | 3 (RB/LB/BB) | 3 | * | * | 3 /13 |

* Not evaluated in the group

**Abbreviations:** BB: RKH Krankenhaus Bietigheim-Bissingen, Germany; ES: Klinikum Esslingen, Esslingen, Germany; F: Die Filderklinik, Filderstadt, Germany; HH: Kreisklinikum Heidenheim, Germany; KA: Städtisches Krankenhaus Karlsruhe, Germany; LB: RKH Kliniken Ludwigsburg, Germany; M: University Medical Center Mannheim, Germany; Ö: Klinik Öschelbronn, Germany; P: Paul-Lechler- Krankenhaus Tübingen, Germany; PU: Paracelsus-Krankenhaus Unterlengenhardt, Germany; RB: Robert Bosch Hospital, Stuttgart, Germany; RM: Rems-Murr Klinikum Winnenden, Germany; UK: Department of General and Visceral Surgery, Section Integrative Medicine, University Hospital Ulm, Germany
