# Supplementary material for: Expert-guided approaches to complementary interventions for common side effects of cancer therapies: a practice-based perspective from integrative oncology centers in Baden-Württemberg, Germany
Source: Front Oncol. 2025 Nov 6;15:1667298. doi: 10.3389/fonc.2025.1667298 (PMC12631479; doi:10.3389/fonc.2025.1667298)
Supplement: Supplementary file 11 [file Table11.docx]

**Supplement 11: Practice Based Recommendation for Chemotherapy-Induced Nausea and Vomiting (CINV)**

**Voting: Best practice recommendations for CINV Consensus**

Total physicians and nurses: 8 P- 6 N - Total institutions: 11

Note to the Table: The "Number of Institutions" and "Effectiveness" columns represent the initial evaluations, as shown in Supplement 7 for physicians and Supplement 8 for nurses. The "Voting X / 10" column shows the final voting results, with each institution casting one vote.

Results - Best Practice Recommendation: (A)-(B)-(C)-(D)-(E)

| **Intervention** | **Physicians** | | **Nurses** | |  |
| --- | --- | --- | --- | --- | --- |
|  | Number of  Institution | Effective-ness | Number of  Institution | Effective-ness | Voting |
| **Therapeutic use** |  |  |  |  |  |
| Acupressure (A) | 7 (RB/F/PU/KA/RM/LB/ BB) | 3 | 4 (RB/F/KA/UK) | 4 | **7 /11** |
| Aromatherapy (B) | 6 (UK/Ö/ES/KA/RB/F) | 4 | 3 (KA/RB/ES) | 4 | **6 /11** |
| Homeopathic preparation (Nux vomica) (C) | 5 (HH/F/B/BB/Ö) | 3 | 2 (Ö/HH) | 3 | **6 /11** |
| Ginger (D) | 7 (RB/F/PU/KA/RM/LB/ BB) | 3 | 2 (RB/F/KA) | 3 | **5 /11** |
| Bitter botanicals (Gentiana lutea) (E) | 9 (HH/F/PU/KA/RB/Ö/LB/BB/UK) | 3 | 1 (UK) | 4 | **6 /11** |
| Wormwood tea | * | * | 2 (HH/KA) | 4 | 2 /11 |
| **Preventive and Therapeutic use** |  |  |  |  |  |
| Mind-Body Medicine | 3 (RB/BB/LB) | 2 | * | * | 4 /11 |

* Not evaluated in the group

Abbreviations: BB: RKH Krankenhaus Bietigheim-Bissingen, Germany; ES: Klinikum Esslingen, Esslingen, Germany; F: Die Filderklinik, Filderstadt, Germany; HH: Kreisklinikum Heidenheim, Germany KA: Städtisches Krankenhaus Karlsruhe, Germany; LB: RKH Kliniken Ludwigsburg, Germany; Ö: Klinik Öschelbronn, Germany; PU: Paracelsus-Krankenhaus Unterlengenhardt, Germany; RB: Robert Bosch Hospital, Stuttgart, Germany; RM: Rems-Murr Klinikum Winnenden, Germany; UK: Department of General and Visceral Surgery, Section Integrative Medicine, University Hospital Ulm, Germany
