# Supplementary material for: Expert-guided approaches to complementary interventions for common side effects of cancer therapies: a practice-based perspective from integrative oncology centers in Baden-Württemberg, Germany
Source: Front Oncol. 2025 Nov 6;15:1667298. doi: 10.3389/fonc.2025.1667298 (PMC12631479; doi:10.3389/fonc.2025.1667298)
Supplement: Supplementary file 12 [file Table12.docx]

**Supplement 12: Targeted (non-systematic) literature research Cancer-Related-Fatigue (CRF)**

|  | **Summarized statement from the targeted (non-systematic) literature research** |
| --- | --- |
| Movement therapy | S3 LL Komp: "Shall recommend" (1-3) |
| Yarrow liver compress | An RCT with positive indications for the use of yarrow liver compresses in patients with metastatic cancer undergoing radiotherapy (5) |
| Viscum album therapy | S3 LL Comp: "may recommend" the use of mistletoe therapy to improve overall quality of life, but no specific recommendation for fatigue (1) |
| Sleep hygiene / circadian rhythm | Comment: Recommendation too general for literature search; no specific studies identified. A systematic review (SR) would be required |
| Hydrotherapy | NEI |

Legend:

S3 LL Komp: S3 guideline on complementary medicine in the treatment of oncology patients

NEI: No Evidence Identified (no relevant publications found in the targeted literature search; inclusion based on clinical consensus or limited preliminary data

Literature:

1. Deutsche Krebsgesellschaft. S3-Leitline Komplementärmedizin in der Behandlung von onkologischen Patienten; 2024. Available from: URL: https://www.awmf.org/leitlinien/detail/ll/032-055OL.html.
2. Bower JE, Lacchetti C, Alici Y, Barton DL, Bruner D, Canin BE et al. Management of Fatigue in Adult Survivors of Cancer: ASCO-Society for Integrative Oncology Guideline Update. J Clin Oncol 2024; 42(20):2456–87.
3. National Comprehensive Cancer Network (NCCN). NCCN Clinical Practice Guidelines in Oncology (NCCN Guidelines®): Cancer-Related Fatigue; 2025. Available from: URL: : https://www.nccn.org/professionals/physician_gls/pdf/fatigue.pdf. Accessed:25.09.2025.
4. hadjar P, Stritter W, von Mackensen I, Mehrhof F, Foucré C, Ehrhardt VH, et al. External application of liver compresses to reduce fatigue in patients with metastatic cancer undergoing radiation therapy, a randomized clinical trial. Radiat Oncol. 2021;16(1):76.
5. Dean R. Can improving quality of sleep reduce the symptoms of cancer-related fatigue in adults?: A systematic review. Eur J Cancer Care (Engl) 2022; 31(4):e13597.
