# Supplementary material for: Expert-guided approaches to complementary interventions for common side effects of cancer therapies: a practice-based perspective from integrative oncology centers in Baden-Württemberg, Germany
Source: Front Oncol. 2025 Nov 6;15:1667298. doi: 10.3389/fonc.2025.1667298 (PMC12631479; doi:10.3389/fonc.2025.1667298)
Supplement: Supplementary file 13 [file Table13.docx]

**Supplement 13: Targeted (non-systematic) literature research - Chemotherapy-Induced-Mucositis (CIM)**

|  | **Summarized statement from the targeted (non-systematic) literature research** |
| --- | --- |
| Herbal tea sage mouthwash | KOKON Network: To date, there are no statements on preparations made from sage leaves in the published guidelines. Despite the limited number of studies, sage tea is nevertheless very important in supportive therapy. Care should be taken to ensure that the preparations are of high pharmaceutical quality. |
| Ice cubes | Indication of low efficacy for prophylaxis from study on clinical practice guidelines (1-4) |
| Sea buckthorn fruit oil - mouth rinses | Indication of very good efficacy for prophylaxis and therapy from study on clinical practice guidelines (1) |
| Frozen pineapple cubes | Indication of good efficacy for therapy from study on clinical practice guidelines (1) |
| Herbal oral balm(WALA Oral Balm ®) containing calendula, myrrh, and ratanhia) | NEI |

Legend:

S3 LL Komp: S3- Guideline on complementary medicine in the treatment of cancer patients

NEI: No Evidence Identified (no relevant publications found in the targeted literature search; inclusion based on clinical consensus or limited preliminary data)

Literature:

1. Steinmann D, Babadağ Savaş B, Felber S, Joy S, Mertens I, Cramer H, et al. Nursing Procedures for the Prevention and Treatment of Mucositis Induced by Cancer Therapies: Clinical Practice Guideline Based on an Interdisciplinary Consensus Process and a Systematic Literature Search. Integr Cancer Ther. (2021);20 <https://doi.org/10.1177/1534735420940412>
2. López-González Á, García-Quintanilla M, Guerrero-Agenjo CM, Tendero JL, Guisado-Requena IM, Rabanales-Sotos J. Eficacy of Cryotherapy in the Prevention of Oral Mucosistis in Adult Patients with Chemotherapy. Int J Environ Res Public Health 2021; 18(3).
3. Soliman HMM. The effect of cryotherapy on chemotherapy induced oral mucositis in Egyptian cancer patients: A randomized controlled trial. JNEP 2019; 9(11):63.
4. Elad S, Cheng KKF, Lalla RV, Yarom N, Hong C, Logan RM et al. MASCC/ISOO clinical practice guidelines for the management of mucositis secondary to cancer therapy. Cancer 2020; 126(19):4423–31.
