# Supplementary material for: Expert-guided approaches to complementary interventions for common side effects of cancer therapies: a practice-based perspective from integrative oncology centers in Baden-Württemberg, Germany
Source: Front Oncol. 2025 Nov 6;15:1667298. doi: 10.3389/fonc.2025.1667298 (PMC12631479; doi:10.3389/fonc.2025.1667298)
Supplement: Supplementary file 14 [file Table14.docx]

**Supplement 14: Targeted (non-systematic) literature research - Nausea and Vomiting**

|  | **Summarized statement from the targeted (non-systematic) literature research** |
| --- | --- |
| Acupressure | S3 LL Comp "can recommend" for acupressure using Pericardium 6 (PC 6). (1) |
| Aromatherapy | S3 LL Comp**:** no information. In LL only statement on aromatherapy massage, no conclusion on efficacy possible there. Recommendation too general for a specific literature search.  A systematic review and meta-analysis of randomized controlled trials. (2) |
| Nux vomica | NEI |
| Ginger | S3 LL Komp "can recommend”. (1) |
| Bitter substances | NEI |

Legend:

S3 LL Komp: S3 guideline on complementary medicine in the treatment of oncology patients

PC 6: Pericardium 6

NEI: No Evidence Identified (no relevant publications found in the targeted literature search; inclusion based on clinical consensus or limited preliminary data)

Literature:

1. Oncology guideline program (German Cancer Society DK, AWMF). S3 guideline Complementary medicine in the treatment of oncology patients (2021); AWMF register number: 032/055OL (long version 1.1,). Available at: [https:// .www.leitlinienprogramm-onkologie.de/leitlinien/komplementaermedizin](https://www.leitlinienprogramm-onkologie.de/leitlinien/komplementaermedizin)
2. Ahn JH, Kim M, Kim RW. Effects of aromatherapy on nausea and vomiting in patients with cancer: A systematic review and meta-analysis of randomized controlled trials. Complement Ther Clin Pract 2024; 55:101838.
