# Supplementary material for: Expert-guided approaches to complementary interventions for common side effects of cancer therapies: a practice-based perspective from integrative oncology centers in Baden-Württemberg, Germany
Source: Front Oncol. 2025 Nov 6;15:1667298. doi: 10.3389/fonc.2025.1667298 (PMC12631479; doi:10.3389/fonc.2025.1667298)
Supplement: Supplementary file 15 [file Table15.docx]

**Supplement 15: Targeted (non-systematic) literature search**

Search **Strategy**

A targeted (non-systematic) literature search was conducted to support the consensus-based recommendations. Searches were carried out in PubMed/Medline, the KOKON knowledge database (Komplementärmedizin in der Onkologie), and the German AWMF S3 Guidelines (Complementary Medicine in Oncology and Supportive Therapy).

**Search domains and terms**

- **Cancer/oncology context:**
  cancer, oncology, neoplasm, tumor, malignancy, chemotherapy, cancer therapy, radiotherapy
- **Symptom domains:**
  - Cancer-related fatigue (CRF): fatigue, cancer-related fatigue, asthenia, tiredness, exhaustion
  - Chemotherapy-induced nausea and vomiting (CINV): nausea, vomiting, emesis, chemotherapy-induced nausea, chemotherapy-induced vomiting
  - Chemotherapy-induced mucositis (CIM): mucositis, oral mucositis, stomatitis, oral toxicity, oral inflammation
- **Interventions (15 top-rated from the consensus process):**
  - CRF: movement therapy/exercise, yarrow liver compress, mistletoe (Viscum album), sleep hygiene/daily rhythm, hydrotherapy
  - CINV: acupressure, aromatherapy, Nux vomica, ginger (Zingiber officinale), gentian (Gentiana lutea)
  - CIM: sage mouthwash (Salvia officinalis), ice cubes/oral cryotherapy, sea buckthorn oil (Hippophae rhamnoides), frozen pineapple cubes (Ananas comosus), herbal oral balm (calendula, myrrh, ratanhia)
- **Broader search terms:**
  To capture studies not explicitly mentioning the intervention in the title or abstract, broader search terms were included, such as:

complementary medicine, complementary and alternative medicine, CAM, integrative oncology, integrative medicine, anthroposophic medicine, naturopathy, phytotherapy, herbal medicine, mind–body therapy, supportive care, lifestyle intervention

Search terms were combined using Boolean operators (AND/OR) to link cancer/oncology terms with the relevant symptom domain and the respective interventions. Equivalent German terms were applied in the KOKON database. Relevant records were screened in a two-step process (title/abstract, then full text). Extracted data were used to complement the expert consensus process. Detailed findings are provided in Supplements 12–14.
